# Early-life ruminal microbiome-derived indole-3-carboxaldehyde and prostaglandin D2 are effective promoters of rumen development

**DOI:** 10.1186/s13059-024-03205-x

**Published:** 2024-03-04

**Authors:** Daming Sun, Gaorui Bian, Kai Zhang, Ning Liu, Yuyang Yin, Yuanlong Hou, Fei Xie, Weiyun Zhu, Shengyong Mao, Junhua Liu

**Affiliations:** 1 Laboratory of Gastrointestinal Microbiology, Jiangsu Key Laboratory of Gastrointestinal Nutrition and Animal Health, National Center for International Research On Animal Gut Nutrition, College of Animal Science and Technology, Nanjing Agricultural University, Nanjing, 210095 China; 2 College of Animal Science and Food Engineering, Jinling Institute of Technology, Nanjing, 210038 China; 3 Huzhou Academy of Agricultural Sciences, Huzhou, 313000 China; 4 Laboratory of Metabolism and Drug Target Discovery, State Key Laboratory of Natural Medicines, College of Pharmacy, China Pharmaceutical University, Nanjing, 210009 China

**Keywords:** Rumen development, Solid diet, Lamb, Microbial–host interaction

## Abstract

**Background:**

The function of diverse ruminal microbes is tightly linked to rumen development and host physiology. The system of ruminal microbes is an excellent model to clarify the fundamental ecological relationships among complex nutrient–microbiome–host interactions. Here, neonatal lambs are introduced to different dietary regimes to investigate the influences of early-life crosstalk between nutrients and microbiome on rumen development.

**Results:**

We find starchy corn-soybean starter-fed lambs exhibit the thickest ruminal epithelia and fiber-rich alfalfa hay-fed lambs have the thickest rumen muscle. Metabolome and metagenome data reveal that indole-3-carboxaldehyde (3-IAld) and prostaglandin D2 (PGD2) are the top characteristic ruminal metabolites associated with ruminal epithelial and muscular development, which depend on the enhanced ruminal microbial synthesis potential of 3-IAld and PGD2. Moreover, microbial culture experiment first demonstrates that *Bifidobacterium pseudolongum* is able to convert tryptophan into 3-IAld and *Candida albicans* is a key producer for PGD2. Transcriptome sequencing of the ruminal epithelia and smooth muscle shows that ruminal epithelial and muscular development is accompanied by Wnt and Ca^2+^ signaling pathway activation. Primary cell cultures further confirm that 3-IAld promotes ruminal epithelial cell proliferation depending on AhR-wnt/β-catenin signaling pathway and PGD2 accelerates ruminal smooth muscle cell proliferation via Ca^2+^ signaling pathway. Furthermore, we find that 3-IAld and PGD2 infusion promote ruminal epithelial and musculature development in lambs.

**Conclusions:**

This study demonstrates that early-life ruminal microbiome-derived 3-IAld and PGD2 are effective promoters of rumen development, which enhances our understanding of nutrient–microbiome–host interactions in early life.

**Supplementary Information:**

The online version contains supplementary material available at 10.1186/s13059-024-03205-x.

## Background

Diet is an indispensable modulator in shaping gastrointestinal microbial communities through nutrient intake and digestion [1]. In this process, microbial metabolites can exert as signaling or substrates to influence the host’s organ development and metabolic health [2, 3]. Although the diet–microbiome–host interaction, which forms a complex symbiont, has attracted great interest in how it can benefit human and animal health, the relationship among them remains largely unknown [3]. To date, the current understanding of the relationship has mostly been derived from monogastric animals [4, 5], while the diet–microbial interaction was limited because of high enzymatic digestion in the foregut [6]. Alternatively, ruminants occupy a much more complex microbial community, conferring host cells that can utilize an extensive amount of nondigestible fibers for body protein and milk production [7]. The high degree of the symbiont is attributed to rumen, the natural fermentation chamber, where diverse and enriched microbes inhabit, including bacteria, archaea, fungi, and ciliated protozoa [8].

In the rumen, microbes not only efficiently convert dietary fibers into metabolic precursors, such as volatile fatty acids (VFA), microbial protein, and vitamins [9], but they also affect rumen stratified epithelial and muscular development [10, 11]. Rumen smooth muscle facilitates microorganisms’ good contact with feeds for digestion, and stratified epithelia can effectively absorb fermented nutrients to influence the host [10]. Hence, the interaction between diets and microbiome is tightly linked to rumen development, functions, and host metabolic health.

The rumen microbial initial colonization and maturation after birth could be highly impacted by varied dietary accessibility, such as milk, grain-derived solid feeds, hays, or a combination of these [12–14]. However, how the postnatal ruminal microbiota enrichment with different dietary nutrient availability alters rumen wall development and animal growth remains elusive. To fill this gap, we used a lamb model to investigate the impacts of early-life cross-talking between nutrients and microbiota on rumen wall development with diverse dietary niches. In the current study, we found that the introduction of a solid diet elevated the conversion of nutrients into bioactive microbial metabolites, which drove development in host rumen epithelia and muscle layer morphology. Specifically, we identified two ruminal microbe-derived metabolites (indole-3-carboxaldehyde, 3-IAld; and prostaglandin D2, PGD2) that promoted rumen epithelial and muscular development, overcoming the limitation of conventional knowledge that mainly focuses on VFA promoting rumen epithelia development and diet physical factors facilitating rumen muscle development.

## Results

### Varied dietary regimes drove early-life disparity in rumen wall development

**Fig. 1:**
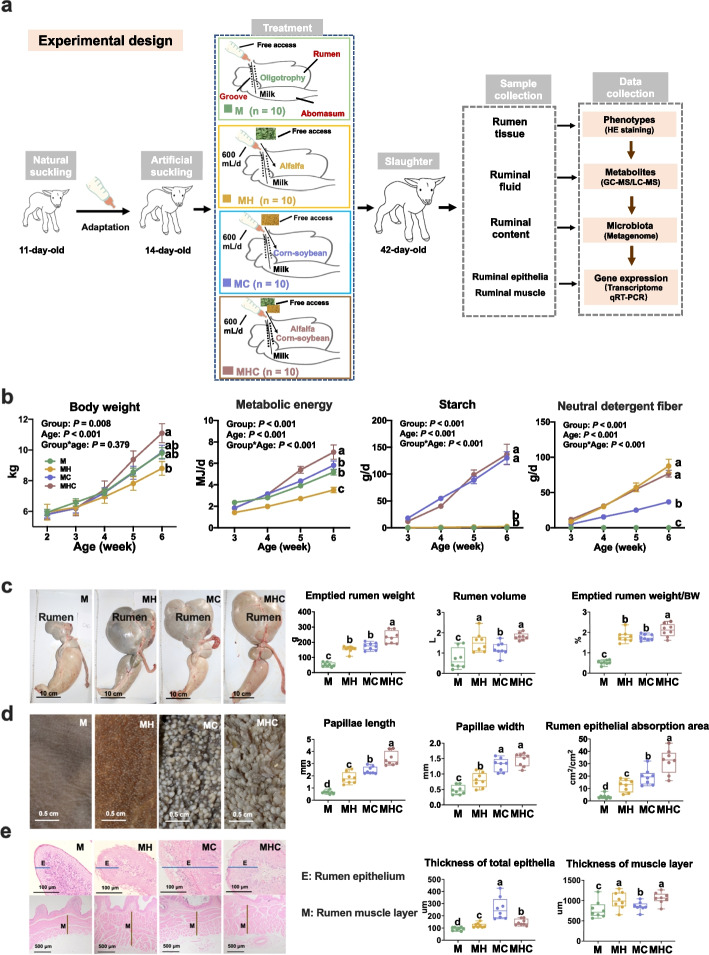
Construction of the varied rumen development phenotypes with extremely different dietary regimes. **a** Experimental study design; Hu lambs were randomly assigned to four groups following diets: milk (M, *n* = 10), milk plus alfalfa hay (MH, *n* = 10), milk plus corn-soybean starter (MC, *n* = 10), and milk plus alfalfa hay and corn-soybean starter (MHC, *n* = 10). **b** Body weight and daily nutrient intake of lambs; the statistical analysis was performed using the mixed linear model. **c–e** Pictures and measured parameters of rumen organ (**c**), rumen papillae (**d**), and rumen wall thickness (**e**). Data represent the mean ± SEM, *n* = 8 per group. Means with different letters were significantly different (*P* < 0.05) via one-way ANOVA, followed by post hoc Tukey tests

Here, we established a lamb model of rumen tissue phenotypes altered by varied early-life dietary regimes (Fig. 1a). The results showed that milk-fed lambs with little starch and fiber intake (Fig. 1b) exhibited the smallest rumen weight and volume, with the thinnest ruminal epithelia and muscle (Fig. 1c–e). Starchy corn-soybean starter or fiber-rich alfalfa hay introduction markedly increased the emptied rumen weight, rumen volume, and ruminal papillae length, width, and surface in lambs (Fig. 1c, d). Specifically, the lambs with starchy corn-soybean starter introduction had the greatest starch intake (Fig. 1b) and thickest rumen epithelia (Fig. 1e); the lambs with alfalfa hay supplementation had the greatest neutral detergent fiber intake (Fig. 1b) and the thickest rumen muscle (Fig. 1e). Compared with corn-soybean starter introduction, cofeeding of corn-soybean starter and alfalfa hay decreased the thickness of rumen epithelia (Fig. 1e), while increased the length of ruminal epithelial papillae (Fig. 1d). Previous study indicated the elongation of gastrointestinal epithelium through regulation cell division, cell elongation, and cell rearrangement [15]. Thus, we speculated that cofeeding of corn-soybean starter and alfalfa hay facilitated the ruminal epithelial cell division orientation for the growth of ruminal epithelial papillae. Overall, starchy corn-soybean starter and fiber-rich alfalfa hay specially promoted ruminal epithelial and muscular development, respectively.

### 3-IAld and PGD2 were the top characteristic ruminal metabolites associated with ruminal epithelial and muscular development

**Fig. 2:**
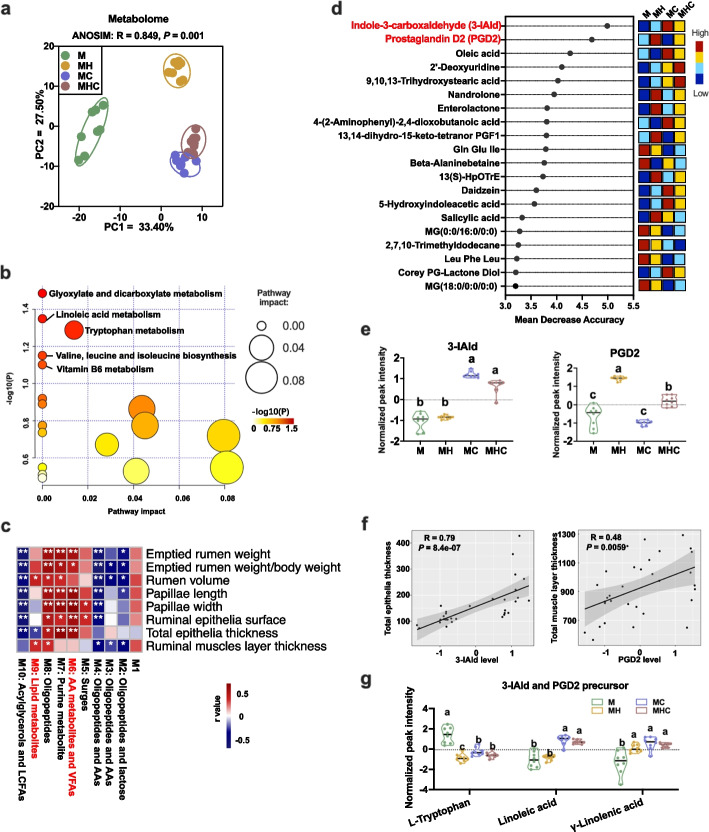
Identification of the ruminal characteristic metabolites with varied dietary regimes. **a** PCA plot of metabolites detected by metabolome in the rumen liquid samples of lambs among four groups. **b** Pathway enrichment analysis was performed using the top 50 significantly (FDR < 0.05) different metabolites among groups, and the difference among the four groups was identified using the Kruskal–Wallis test (*n* = 8 per group). **c** WGCNA identification of rumen metabolite modules correlated with rumen development indexes, *P < 0.05, **P < 0.001. **d** The top 20 metabolites with the strongest influence on prediction accuracy of the random forest analysis presented in order of importance (top to bottom); the colored boxes on the right represented the relative level of the corresponding metabolites in each group. **e** The levels of 3-IAld and PGD2 in the rumen among four groups; means with different letters were significantly different via comparing any two diets by a fold-change threshold of 2 and a false discovery rate (FDR) < 0.05 from Wilcoxon rank-sum test and Benjamini and Hochberg multiple testing corrections. **f** Spearman’s correlation analysis between levels of 3-IAld and PGD2 and thickness of rumen epithelia and muscle layer. **g** The level of 3-IAld and PGD2 precursors in the rumen fluid; means with different letters were significantly different via comparing any two diets by a fold-change threshold of 2 and a false discovery rate (FDR) < 0.05 from Wilcoxon rank-sum test and Benjamini and Hochberg multiple testing corrections

Next, we raised the question of what changes in ruminal metabolic pathways contributed to the disparity of rumen epithelial and muscular development. Rumen liquid samples were analyzed by untargeted gas chromatography–mass spectrometry (GC–MS) and ultraperformance liquid chromatography coupled with quadrupole time-of-flight mass spectrometry (UPLC-Q-TOF/MS). Principal component analysis (PCA) of annotated metabolites showed a qualitative difference among the four groups (Fig. 2a). Next, we enriched the top 50 significantly different metabolites among groups based on the Kyoto Encyclopedia of Genes and Genomes (KEGG) pathways. The top pathway terms were related to tryptophan metabolism and linoleic acid metabolism (Fig. 2b). From the heat map of the top 50 significantly different metabolites among groups, we found that corn-soybean starter introduction specially increased the level of indole derivatives (tryptophan metabolites) and alfalfa hay specially elevated the level of prostaglandins (arachidonic acid metabolites) (Additional file 1: Fig. S1). To determine the specific metabolites related to rumen epithelia and muscle development index, all metabolites were binned into 10 co-abundance clusters by weighted gene co-expression network analysis (WGCNA). Here, amino acid metabolites and VFAs (M6, including 3-IAld and butyrate) were especially positively linked to rumen epithelial developmental index; Lipid metabolites (M9, including PGD2 and PGD3) were especially positively associated with rumen muscular developmental index (Fig. 2c; Additional file 2: Table S1). Moreover, we performed a random forest classifier using the level of metabolites as variables (100% classification accuracy). Random forest result further indicated that 3-IAld was the top characteristic ruminal metabolite in lambs fed corn-soybean starter (Fig. 2d, e), and the 3-IAld level was positively correlated with the thickness of rumen epithelia (Fig. 2f). The level of 3-IAld precursor tryptophan was highest in lambs with only milk intake (Fig. 2g). Moreover, PGD2 was the top characteristic ruminal metabolite of alfalfa hay introduction (Fig. 2d, e). Importantly, a positive correlation of ruminal PGD2 level with rumen muscle layer thickness was observed (Fig. 2f). Meanwhile, lambs with corn-soybean starter introduction exhibited the highest level of ruminal linoleic acid and gamma-linolenic acid (Fig. 2g). Together, 3-IAld and PGD2 were the top characteristic ruminal metabolites associated with ruminal epithelial and muscular development.

### Clarification of the ruminal symbiotic microbial metabolic pathways during 3-IAld and PGD2 synthesis

**Fig. 3:**
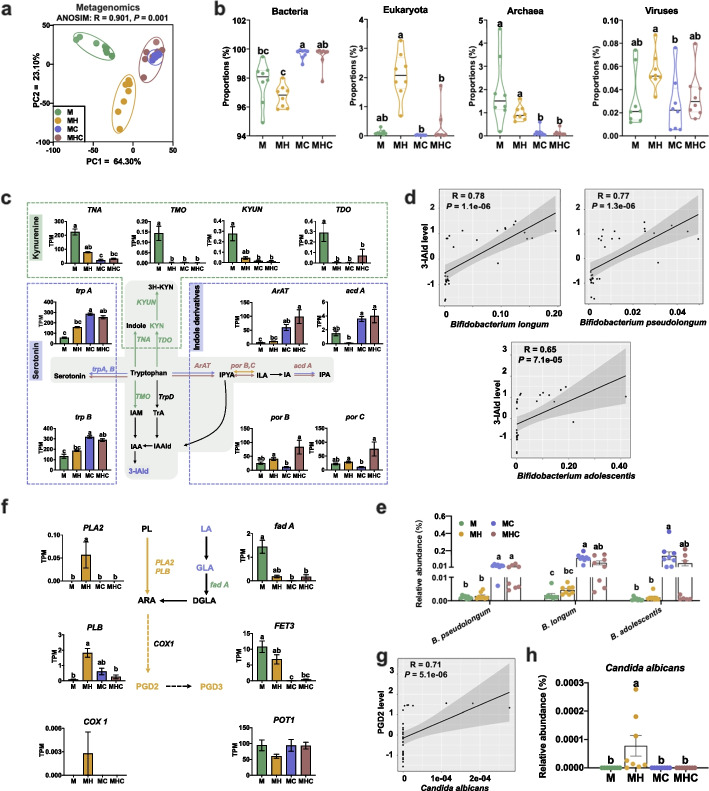
Clarification of the ruminal symbiotic microbial metabolic pathway during 3-IAld and PGD2 synthesis. **a** PCA analysis of metagenomic data based on KEGG functional database (*n* = 8). **b** Comparison of microbial domains in the rumen contents of lambs among four groups (*n* = 8); statistical analysis was performed using the Kruskal–Wallis test, with the FDR < 0.05 being regarded as significantly different. **c** Pathways of tryptophan metabolism and 3-IAld generation in the ruminal microbiota. A green word or line represented the highest gene abundance in the M group, yellow represented MH, blue represented MC, and brown represented MHC group. **d** Spearman’s correlation analysis (*P* < 0.05, |*r*|> 0.65) between the relative abundance of microbial species and 3-IAld level in the rumen. **e** The relative abundance of *Bifidobacterium* species related to tryptophan metabolism. **f** Pathways of arachidonic acid metabolism and PGD2 generation in the ruminal microbiota; A green word or line represented the highest gene abundance in M group, yellow represented MH, blue represented MC, and brown represented MHC group. **g** Spearman’s correlation analysis (*P* < 0.05, |*r*|> 0.65) between the relative abundance of *Candida albicans* and PGD2 level in the rumen. **h** The relative abundance of *Candida albicans* in the rumen. Statistical analysis was performed using the Kruskal–Wallis test, with the FDR < 0.05 being regarded as significantly different (*n* = 8). 3H-KYN: 3-Hydroxykynurenine; KYN: Kynurenine; *acdA*: *Acyl-CoA dehydrogenase*; ARA: Arachidonic acid; *ARAT*: *Aromatic amino acid aminotransferase*; *COX1*: *Cyclooxygenase 1*; DGLA: Dihomo-gamma-linolenic acid; fadA: Acetyl-CoA acyltransferase; GLA: Gamma-linolenic acid; IAA: Indole acetic acid; IAAld: Indole-3-acetaldehyde; 3-IAld: Indole-3-carboxaldehyde; IAM: Indole-3-acetamide; ILA: Indole-3-lactic acid; IPA: Indole-3-propionic acid; IPYA: Indole-3-pyruvate; *KYNU*: *Kynureninase*; LA: Linolenic acid; PGD: Prostaglandin D; *porB, C*: *pyruvate ferredoxin oxidoreductase B and C*; PL: Phospholipids; *PLA*, *B*: Phospholipase A and B; *TDO*: *Tryptophan 2,3-Dioxygenase*; *TMO*: *Tryptophan 2-monooxygenase*; *TNA*: *Tryptophanase*; *TRA*: *Tryptamine; trpA, B*: *tryptophan synthase alpha and beta chain*

To evaluate the ruminal microbial synthesis potential of 3-IAld and PGD2, we conducted analysis of metagenomic data sets. Metagenome sequencing of 32 rumen contents samples generated a total of 11,142.8 million raw reads pairs, and 8732.3 million reads pairs passed through quality filtering. We generated a nonredundant rumen microbial gene catalog that comprises 12,707,419 genes. According to currently available databases, 48.93% of the rumen microbial gene catalog were taxonomically classified as originating from bacteria (47.21%), archaea (1.02%), eukaryote (0.58%) and virus (0.04%), and 41.87% (5,320,684) of the rumen microbial gene catalog were annotated to KEGG orthologous groups (KOs). PCA analysis was performed based on all detected KEGG orthology and revealed that there was a significant difference among four groups (Fig. 3a). We observed that corn-soybean-fed lambs were frequently enriched with ruminal bacteria, which may just be the reflection of the depletion of ruminal archaea, while alfalfa hay-fed lambs were more occupied with eukaryota in the rumen (Fig. 3b). Striking differences were found in genes related to the formation of indole, kynurenine, serotonin, and indole derivatives via microbial metabolism (Fig. 3c). The indolepyruvate route was one of the main pathways for 3-IAld synthesis from tryptophan and was catalyzed by the *aromatic amino acid aminotransferase* (*ArAT*) [16]. Our results showed that starchy corn-soybean starter-fed lambs elevated the abundance of *ArAT* (Fig. 3c). We next checked the bacterial genus and species previously reported as tryptophan metabolism [17]. Noticeably, the ruminal 3-IAld level was positively correlated with the relative abundance of three ruminal *Bifidobacterium* species, including *Bifidobacterium adolescentis*, *Bifidobacterium longum*, and *Bifidobacterium pseudolongum* (Fig. 3d, e).

Our study also revealed that fiber-rich alfalfa hay facilitated the enrichment of ruminal eukaryotes in lambs. Substantial evidence has demonstrated that gastrointestinal fungi had the ability to produce prostaglandins from arachidonic acid [18–26]. Notably, alfalfa hay-fed lambs exhibited the highest abundance of microbial enzyme gene *phospholipase A2* (*PLA2*) and *phospholipase B* (*PLB*) (Fig. 3f), which played important roles in the conversion of phospholipids into arachidonic acid [24]. However, detailed biosynthetic pathways of PGD2 from arachidonic acid are still unavailable in fungi. Moreover, we detected the fungal species reported as producing PGD2 or a precursor based on the accumulating previous studies [18, 25, 27–29]. We found the ruminal PGD2 level was positively related to the relative abundance of ruminal *Candida albicans* (Fig. 3g, h).

### Bifidobacterium pseudolongum and Candida albicans had the ability to produce 3-IAld and PGD2, respectively

**Fig. 4:**
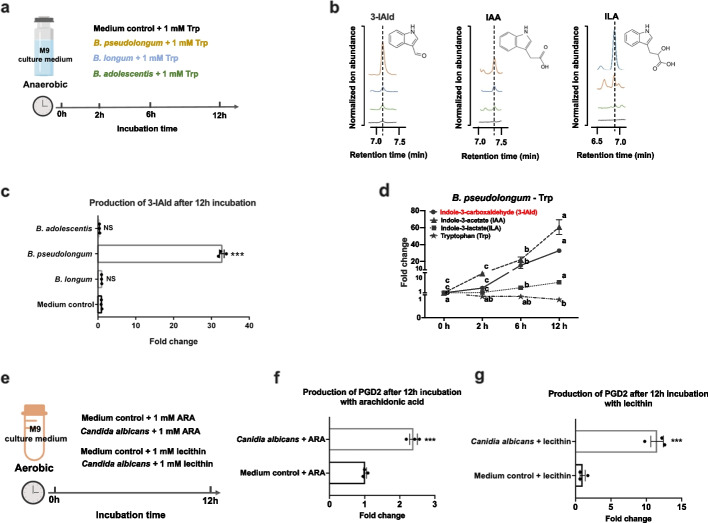
Confirmation of the key microbial producer of 3-IAld and PGD2. **a** Schematic of experimental design of *Bifidobacterium* species incubation in vitro. **b, c ***Bifidobacterium* species were cultured 12 h and assayed for tryptophan metabolites by HPLC in vitro. **d ***Bifidobacterium pseudolongum* produced a wide array of tryptophan-derived metabolites. **e** Schematic of experimental design of *Candida albicans* incubation in vitro. **f, g ***Candida albicans* were cultured 12 h in addition of arachidonic acid (**f**) or lecithin medium (**g**) and assayed for PGD2 metabolites by HPLC in vitro. Data represented the mean ± SEM, *n* = 3 per group. Statistical analysis was performed using independent samples *t*-test (**c**, **f**, and **g**), ***P < 0.001. Means with different letters were significantly different (*P* < 0.05) via ANOVA, followed by post hoc Tukey tests (**d**)

To confirm the capability of these *Bifidobacterium* species to convert tryptophan into 3-IAld, we performed microbial culture experiment in the M9 minimal medium addition of tryptophan (Fig. 4a). The results showed the level of 3-IAld was significantly increased after 12 h incubation with *Bifidobucterium pseudolongum*, suggesting that *Bifidobucterium pseudolongum* was able to convert tryptophan into 3-IAld (Fig. 4b, c). Then, we detected temporal changes in the level of tryptophan, 3-IAld, and other indole derivatives with *Bifidobucterium pseudolongum* inoculation. We found that the level of 3-IAld, indole-3-acetate (IAA), and indole-3-lactate (ILA) gradually increased after *Bifidobucterium pseudolongam* inoculation, while the level of tryptophan gradually decreased (Fig. 4d). As far as we know, this is the first to report that *Bifidobacterium pseudolongum* can metabolize tryptophan into 3-IAld. Similarly, we verified the capability of *Candida albicans* to convert arachidonic acid and lecithin into PGD2 in vitro (Fig. 4e). The results showed the level of PGD2 was significantly elevated after 12 h incubation with *Candida albicans* in the M9 medium addition of arachidonic acid (Fig. 4f) or lecithin (Fig. 4g), supporting that *Candida albicans* was the key producer of PGD2. Therefore, we confirmed that *Bifidobacterium pseudolongum* and *Candida albicans* had the ability to produce 3-IAld and PGD2, respectively.

### Ruminal epithelial and muscular development were accompanied by Wnt and Ca^2+^ signaling pathway activation

To further clarify how the morphological changes in rumen epithelia and muscle were associated with gene regulation, we performed transcriptome sequencing of the ruminal epithelia and smooth muscle. A total of 1027.93 million (43.83 ± 1.55 million reads per sample) and 1156.81 million (48.20 million reads per sample) high-quality, paired reads were generated from ruminal epithelial and muscular samples, respectively. For the rumen epithelial transcriptome, we performed differential gene expression analysis. A total of 778, 226, and 70 differentially expressed genes (DEGs) were obtained in MC versus MH, MHC versus MC, and MHC versus MH, respectively (Additional file 1: Fig. S2). KEGG pathway analyses recovered these DEGs of each comparison were mainly related to metabolism pathways (Additional file 1: Fig. S3). Based on the functional KEGG pathways enrichment in upregulated and downregulated genes for each pairwise comparison between MC, MH, and MHC, we found that starchy corn-soybean starter introduction mainly enriched the rumen epithelial metabolism pathways, biosynthesis of amino acid, and glycerolipid metabolism (Additional file 1: Fig. S4), suggesting that rumen epithelial development was accompanied by enhancement of metabolic function. For the ruminal muscle transcriptome, a total of 140, 131, and 80 DEGs were obtained in MC versus MH, MHC versus MC, and MHC versus MH, respectively (Additional file 1: Fig. S2). Based on the KEGG analysis of DEGs in each comparison, we found that calcium signaling pathway and axon guidance were mainly enriched in these DEGs (Additional file 1: Fig. S5). In addition, we also analyzed the functional KEGG pathways enriched in upregulated and downregulated genes for each pairwise comparison between MC, MH, and MHC and found that alfalfa hay introduction mainly enhanced calcium signaling pathway, adipocytokine signaling pathway, and PPAR signaling pathway (Additional file 1: Fig. S6).

**Fig. 5:**
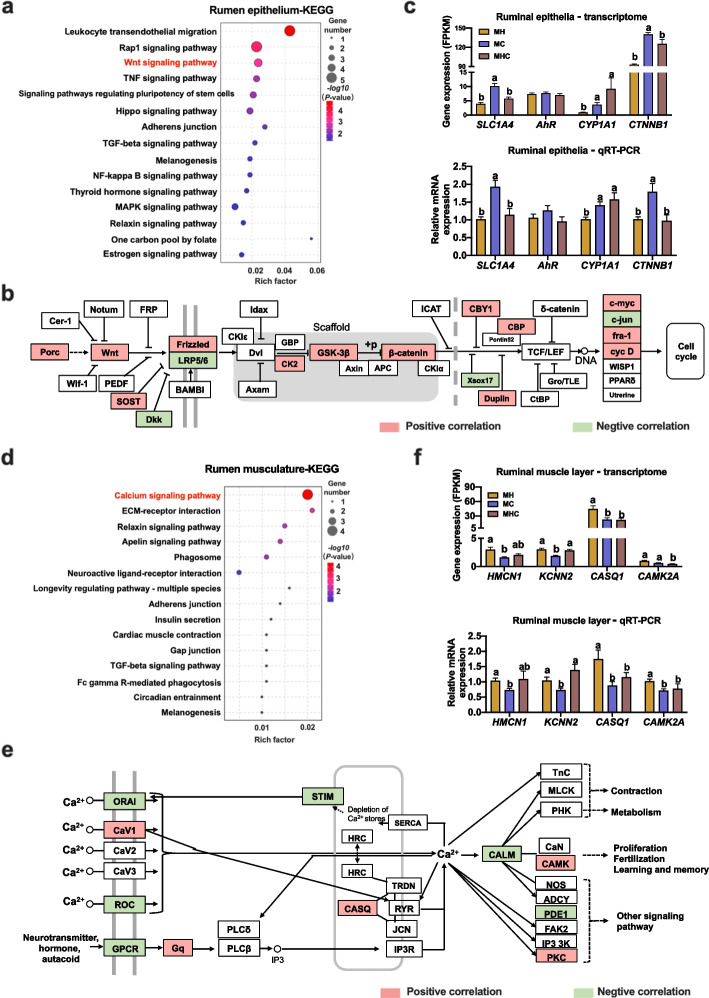
Investigation of the key signaling pathway activation associated with ruminal epithelial and muscular development. **a** Top 15 KEGG enrichment pathway of DEGs associated with ruminal epithelial development in the rumen epithelial samples. **b** Spearman’s correlation analysis (FDR < 0.10) of ruminal epithelial thickness and gene expression related to the Wnt/β-catenin signaling pathway; genes in pathway were marked with red color (positive correlation) or green color (negative correlation) using KEGG mapper tool. **c** The expression of DEGs (|FC|> 1.5 and FDR < 0.05) in ruminal epithelia via qRT-PCR verification (ANOVA). **d** Top 15 KEGG enrichment pathways of DEGs associated with rumen muscle development in the ruminal muscle layer samples. **e** Spearman’s correlation analysis (FD*R* < 0.10) of ruminal muscle layer thickness and gene expression related to the Ca^2+^ signaling pathway; genes in pathway were marked with red color (positive correlation) or green color (negative correlation) using KEGG mapper tool. **f** Expression of DEGs (|FC|> 1.5 and FDR < 0.05) in the ruminal muscle layer via qRT-PCR verification (ANOVA)

Based on all the KEGG analysis for each pairwise comparison between MC, MH, and MHC (Additional file 1: Fig. S3-S6), it was unable to discriminate the specific signal pathways associated with rumen epithelial and muscular development. Here, Gene Ontology (GO) biological processes enrichment analysis was conducted using 1017 DEGs obtained among three groups in the rumen epithelia (Additional file 3: Table S2). We found that epithelium development (GO:0060429), regulation of epithelial cell proliferation (GO:0050678), epithelial cell differentiation (GO:0030855), epithelial cell development (GO:0002064), negative regulation of epithelial cell proliferation (GO:0050680), and epithelial cell proliferation (GO:0050673) were the significantly enriched biological process related to rumen epithelial development (Additional file 4: Table S3). Furthermore, these specific 70 DEGs belonging to biological process of epithelial development and cell proliferation (Additional file 4: Table S3) were selected and clustered for further KEGG analysis. It exhibited that these 70 DEGs were significantly enriched in the leukocyte transendothelial migration, Rap1 signaling pathway, and Wnt signaling pathway (Fig. 5a). The leukocyte transendothelial migration was widely recognized as the key elements of both innate and adaptive immunity of the vessel wall and the underlying tissue [30]. The Rap1 exerted the main function in induction of cell adhesion, either to other cells or to extracellular matrix [31, 32]. Wnt signaling pathway has been demonstrated to play important roles in gut epithelial development and maintaining the proliferative compartment of the adult gut epithelium in multicellular animals [33]. Inspiringly, we investigated the role of Wnt pathway in rumen epithelial development and analyzed the correlation between ruminal epithelial thickness and gene expression related to the Wnt signaling pathway. We found that many key gene expressions were significantly associated with ruminal epithelial development phenotype, such as *Wnt*, *frizzled*, *β-catenin*, and *CCND* (Fig. 5b, Additional file 5: Table S4). Moreover, the expression of *SLC1A4*, *AhR*, *CYP1A1*, and *CTNNB1* was validated by qRT-PCR (Fig. 5c), suggesting that the Wnt/β-catenin signaling pathway could be a candidate pathway involved in ruminal epithelial development.

To further discern the key pathways associated with rumen muscle development, a total of 291 DEGs in the rumen muscle obtained among three groups (Additional file 1: Fig. S2) were used to conduct GO biological processes enrichment analysis (Additional file 6: Table S5). We found that muscle contraction (GO:0006936), connective tissue development (GO:0061448), muscle system process (GO:0003012), smooth muscle contraction (GO:0006939), and muscle structure development (GO:0061061) were the significantly enriched biological process related to rumen muscular development (Additional file 7: Table S6). These specific 26 DEGs belonging to biological process of rumen muscular development (Additional file 7: Table S6) were selected and clustered for further KEGG analysis and it showed that the Ca^2+^ signaling pathway was the top significantly enriched pathway (Fig. 5d). Further analysis showed that many key genes expressed in the Ca^2+^ signaling pathway were significantly correlated with ruminal muscle layer thickness, such as *CAV1*, *CASQ*, and *CAMK* (Fig. 5e, Additional file 8: Table S7). Moreover, the mRNA expressions of *HMCN1*, *KCNN2*, *CASQ1*, and *CAMK2A* were validated using qRT-PCR, and the expression trends remained consistent (Fig. 5f). Therefore, the regulation role of the Ca^2+^ signaling pathway in ruminal muscular development needed to be further clarified.

### 3-IAld and PGD2 accelerated the cell proliferation of ruminal epithelia and smooth muscle via the AhR-Wnt/β-catenin and Ca^2+^/CAMK2 signaling pathways

**Fig. 6:**
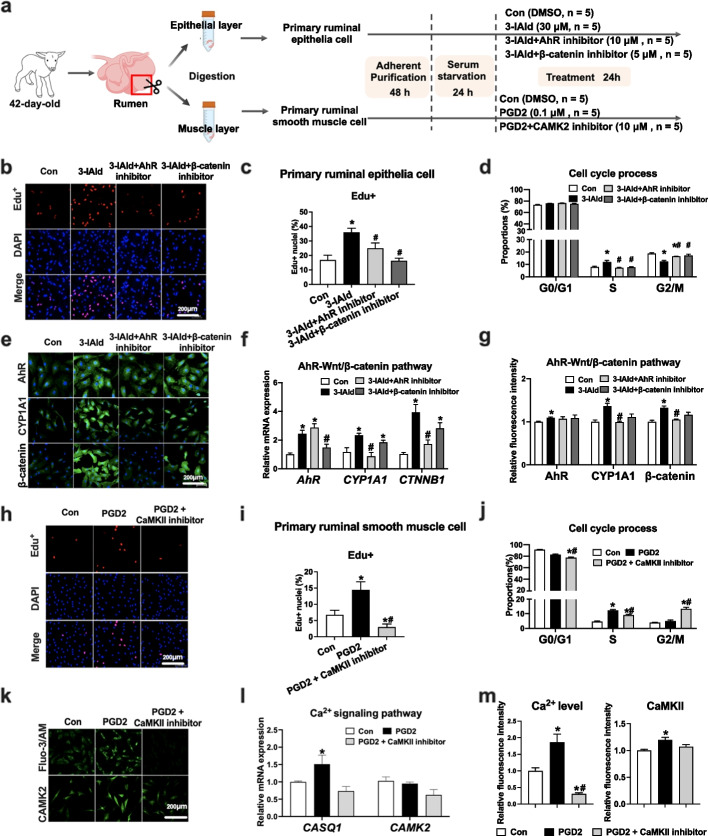
Exploration of the underlying mechanism by which 3-IAld and PGD2 promote rumen epithelial and muscular development. **a** Schematic of cell culture experimental design. Primary ruminal epithelia cells and smooth muscle cells were isolated from the rumen epithelia and muscular layer of Hu lambs (42 days of age). Primary ruminal epithelia cells were divided into control (*n* = 5), 3-IAld (*n* = 5), 3-IAld + AhR inhibitor (*n* = 5), and 3-IAld + β-catenin inhibitor (*n* = 5) group. To identify the effect of PGD2 on primary ruminal smooth muscle cell proliferation, the cells were treated with DMSO (*n* = 5), PGD2 without (*n* = 5) or with CAMK2 inhibitor (*n* = 5). **b, c** 3-IAld increased the proliferation of primary rumen epithelia cells in a AhR-Wnt/β-catenin pathway-dependent manner. **d** 3-IAld accelerated the cell cycle process of primary rumen epithelia cells via AhR-Wnt/β-catenin pathway-dependent manner. **e–g** 3-IAld enhanced the relative mRNA (**f**) and protein (**e** and **g**) levels of *AhR*, *CYP1A1*, and *β-catenin* in the primary rumen epithelia cells. **h, i** PGD2 accelerated proliferation of primary smooth muscle cells in a Ca^2+^ pathway-dependent manner. **j** PGD2 accelerated the cell cycle process of primary rumen smooth muscle cells in a Ca^2+^ pathway-dependent manner. **k, l** PGD2 enhanced the relative mRNA and protein expression of *CASQ1* in the primary rumen smooth muscle cells. **m** PGD2 enhanced intracellular Ca^2+^ level and protein levels of CAMK2 in the primary rumen smooth muscle cells. Data represented the mean ± SEM, *n* = 5 per group. *P < 0.05 compared with the control group; #* P* < 0.05 compared with the 3-IAld or PGD2 group via independent sample *t*-test

According to the above results, we hypothesized that 3-IAld would enable the promotion of ruminal epithelial development via the Wnt/β-catenin signaling pathway. To test this hypothesis, primary rumen epithelia cells were isolated and then treated with 3-IAld (Fig. 6a). In vitro treatment of 3-IAld increased the proliferation of primary rumen epithelia cells, as reflected by increased 5-Ethynyl-2′-deoxyuridine^+^ (EdU^+^) cell ratio (Fig. 6b, c). The upregulation of *CCND1*, *CCNE*, and *CCNB1* was observed with 3-IAld treatment (Additional file 1: Fig. S7a), which was consistent with an acceleration of the cell cycle at the S phase and reduction of the cell cycle arrest at the G2/M phase (Fig. 6d). Meanwhile, 3-IAld enhanced the relative mRNA and protein levels of AhR and β-catenin in the primary ruminal epithelial cells (Fig. 6e–g). We further observed that pretreatment with the AhR inhibitor (CH 223191) or β-catenin inhibitor (XAV939) remarkably attenuated the function of 3-IAld (Fig. 6b, c). Overall, 3-IAld, here as increased by corn-soybean starter feeding, accelerated the proliferation of primary ruminal epithelial cells through an AhR-Wnt/β-catenin pathway-dependent manner.

Then, we further verify whether PGD2 promoted primary ruminal muscular cell proliferation via Ca^2+^ signaling pathway activation (Fig. 6a). We observed that PGD2 treatment increased EdU^+^ cell numbers (Fig. 6h, i) and intracellular Ca^2+^ level (Fig. 6l, m). CaMK2, an important mediator of Ca^2+^ signal, was also upregulated with PGD2 treatment at the mRNA and protein levels (Fig. 6k–m), which regulated the G2/M transition and mitotic progression in mammalian systems [34]. We found that the deletion of CaMK2 significantly inhibited the relative expression of *CCND1*, *CCNA*, and *CCNB1* (Additional file 1: Fig. S7b), which caused cell cycle arrest in G2/M phase (Fig. 6j) and reduced smooth muscle cell proliferation (Fig. 6h, i). Interestingly, the inhibition of CAMK2 not only attenuated the PGD2-induced increase of cell proliferation, but it also inhibited the normal proliferation of cells (Fig. 6h, i). This confirmed that the Ca^2+^/CAMK2 signaling pathway played a crucial role in the proliferation of ruminal smooth muscular cells.

### Infusions of 3-IAld and PGD2 into the rumen of only milk-fed lambs promoted ruminal epithelial and muscular development

**Fig. 7:**
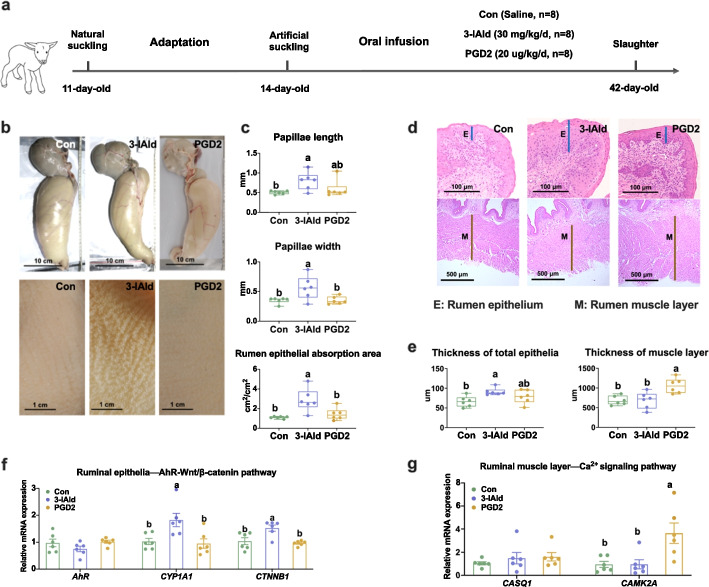
Verification of the effects of 3-IAld and PGD2 infusion on rumen epithelial and muscular development in vivo. **a** Schematic of experimental design. The Hu lambs (11 days of age) were separated from their dams and fed mixed goat milk using an artificial nursing bottle. After 3 days of adaptation, 24 lambs were randomly assigned to three groups with the following treatments: normal saline infusion (Con, *n* = 8), 3-IAld infusion (3-IAld, *n* = 8), and PGD2 infusion (PGD2, *n* = 8). At 42 days of age, the lambs were slaughtered and the ruminal epithelium and muscle tissue were collected for analysis. **b** Representative pictures of the rumen and epithelia at 42 days of age in the study. **c** Effect of 3-IAld and PGD2 infusion on rumen epithelia thickness in only milk-fed lambs. **d** Representative hematoxylin and eosin staining images of the rumen epithelia and muscular layer. **e** Effect of 3-IAld and PGD2 infusion on thickness of rumen epithelia and rumen muscle layer in only milk-fed lambs. **f** Effect of 3-IAld and PGD2 infusion on expression of genes involved in AhR-Wnt/β-catenin signaling pathways in the rumen epithelia of only milk-fed lambs. **g** Effect of 3-IAld and PGD2 infusion on expression of genes involved in Ca^2+^ signaling pathways in the rumen muscle of only milk-fed lambs. Data represented the mean ± SEM, *n* = 6 per group. Means with different letters were significantly different (*P* < 0.05) via one-way ANOVA followed by post hoc Tukey tests

Finally, we aimed to assess the effects of 3-IAld and PGD2 infusion into rumen on ruminal epithelial and muscular development in early life (age 2 to 6 weeks) of only milk-fed lambs (Fig. 7a). Although 3-IAld or PGD2 infusion did not change the weight and volume of the rumen, 3-IAld infusion elevated the rumen papillae length and width, rumen epithelial absorption area, and rumen epithelial thickness (Fig. 7b–e), while PGD2 infusion induced the thicker rumen muscle layer (Fig. 7d, e). Consistent with the in vitro findings, 3-IAld infusion increased the mRNA expression of *CCND1* (Additional file 1: Fig. S7c). PGD2 infusion also accelerated the G1-to-S phase transition of rumen muscle cells, as reflected by the increased mRNA expression of *CCNE*, which is necessary for the orderly completion of the G1-to-S phase transition (Additional file 1: Fig. S7d). 3-IAld infusion also elevated *CYP1A1* and *CTNNB1* mRNA expression in the rumen epithelia (Fig. 7f). Similarly, PGD2 infusion increased *CAMK2A* mRNA expression in the rumen muscular layer (Fig. 7g). Taken together, these data further confirmed that 3-IAld and PGD2 were effective promoters of ruminal epithelial and muscular development.

## Discussion

To date, whether a microbiome exists in the fetal gastrointestinal tract is still controversial, but most scientists agree with the concept of a “sterile womb paradigm” before delivery [35–37]. After birth, the maternal contact and environmental sources contribute to the rumen microbial initial colonization [38] and exert profound life-long effect on rumen development and animal health [39]. Here, solid diet contact affected the rumen microbial colonization and the different nutritional substrate availability also determined ruminal microbial enrichment. Starchy corn-soybean starter encouraged *Bifidobacterium* enrichment, which not only had the characteristics of starch degradation [40, 41], but also the capacity to consume aromatic amino acids. We further confirmed that *Bifidobacterium pseudolongum* was able to convert tryptophan to 3-IAld. The main energy source of *Bifidobacterium* species was the fermentation of a wide range of oligosaccharide and starch [42, 43].

In the rumen of alfalfa hay feeding lambs, the fungi occupied a unique niche. Similarly, the high abundance of fungi in the rumen not only enhanced the ability of fiber degradation, but also increased the opportunity for the metabolism of other substances, such as polyunsaturated or saturated fatty acids [18, 25, 27]. In the present study, lambs supplemented with fiber-rich alfalfa hay continuously enriched their fungal abundance, establishing a microbial environment with a high abundance of fungi and one conducive to the production of PGD2. A previous study reported that *PLA2* and *PLB*, which are released by fungi, play important roles in the conversion of phospholipids into arachidonic acid [24]. However, detailed biosynthetic pathways of PGD2 from arachidonic acid are still unavailable in fungi. In the mammalian cells, cyclooxygenase (COX) enzymatic pathway catalyzes the rate-limiting step in conversion of arachidonic acid into PGD2 [23]. There seems to be a causal relationship between COX and fungi PG production since inhibition of COX enzyme activity reduced PGE2 and PGD2 production in several fungus [44]. In the present study, the expression of COX1 was only detected in alfalfa hay-fed lambs. This study further confirmed that higher relative abundance of *Candida albicans* with alfalfa hay introduction encouraged phospholipids and arachidonic acid to convert into PGD2. Although *Candida albicans* was considered a pathogen in intestine [18], there was no reports of its pathogenicity in the rumen. Therefore, its impact on the host may have two sides, but the threshold value still need to be further explored.

Despite the widely recognized importance of microbial metabolites in the crosstalk between gut microbiota and the host, mechanistic insights into the contribution of ruminal microbe-derived metabolites to rumen development were mainly focused on VFAs [45, 46]. Intriguingly, our research has confirmed that 3-IAld favors rumen epithelia development via the AhR-Wnt/β-catenin signaling pathway. These findings coincide with the paramount role of rumen microbial metabolites in driving rumen development, here adding 3-IAld to this list of signaling metabolites. Earlier research indicated that physical stimulation by fiber-rich diet was the main factor in increasing rumen volume and musculature [45]. Our study has been the first to demonstrate that the ruminal microbe-derived metabolite PGD2 exerted a profound influence on rumen muscle growth via an intracellular Ca^2+^ signaling cascade. Thinkingly, fungal synthesis of PGD2 promoted the development and motility of rumen muscle, which also partially explained why yeast cultures have a good effect on preventing acidosis and maintaining rumen health in ruminant production [47].

Here, we have gained some notable findings, but due to the limitations of agriculture animal feeding and analysis technology, some issues still remain to be further elucidated. First, we did not provide direct evidence to confirm the effect of *Bifidobacterium pseudolongum* and *Candida albicans* on rumen development. The live *Bifidobacterium pseudolongum* and *Candida albicans* oral infusion experiment are required in vivo. Second, in order to truly link microbial species to the metabolism of 3-IAld and PGD2, the growth of candidate microbes with their epithelium/muscle cell cultures without any initial presence of 3-IAld and PGD2 is needed, although it is difficult to preclude the impacts derived from 3-IAld and PGD2 or other metabolites. Third, our study demonstrated that *Bifidobacterium pseudolongum* was able to convert tryptophan into 3-IAld and *Candida albicans* was a key producer for PGD2, but the key conversion pathway and microbial enzyme gene involved in this process need to be further clarified. Moreover, this study added microbial metabolite 3-IAld to the list of effective promoters of rumen epithelial development. However, we did not compare the different effect between 3-IAld and butyrate, which is always considered to be the main ruminal microbial metabolite promoting rumen epithelial development [45]. Nevertheless, it seems that the effects of 3-IAld and PGD2 on gut epithelial and muscle tissues have been previously established [48, 49], but the rumen stratified squamous epithelium structure differ from gut simple columnar epithelium and represent special microbial niche compartmentalization. Lastly, except for ruminal 3-IAld and PGD2, the contributions to rumen development of other characteristic ruminal metabolites related to corn-soybean starter or alfalfa hay introduction need to be further explored.

## Conclusions

**Fig. 8:**
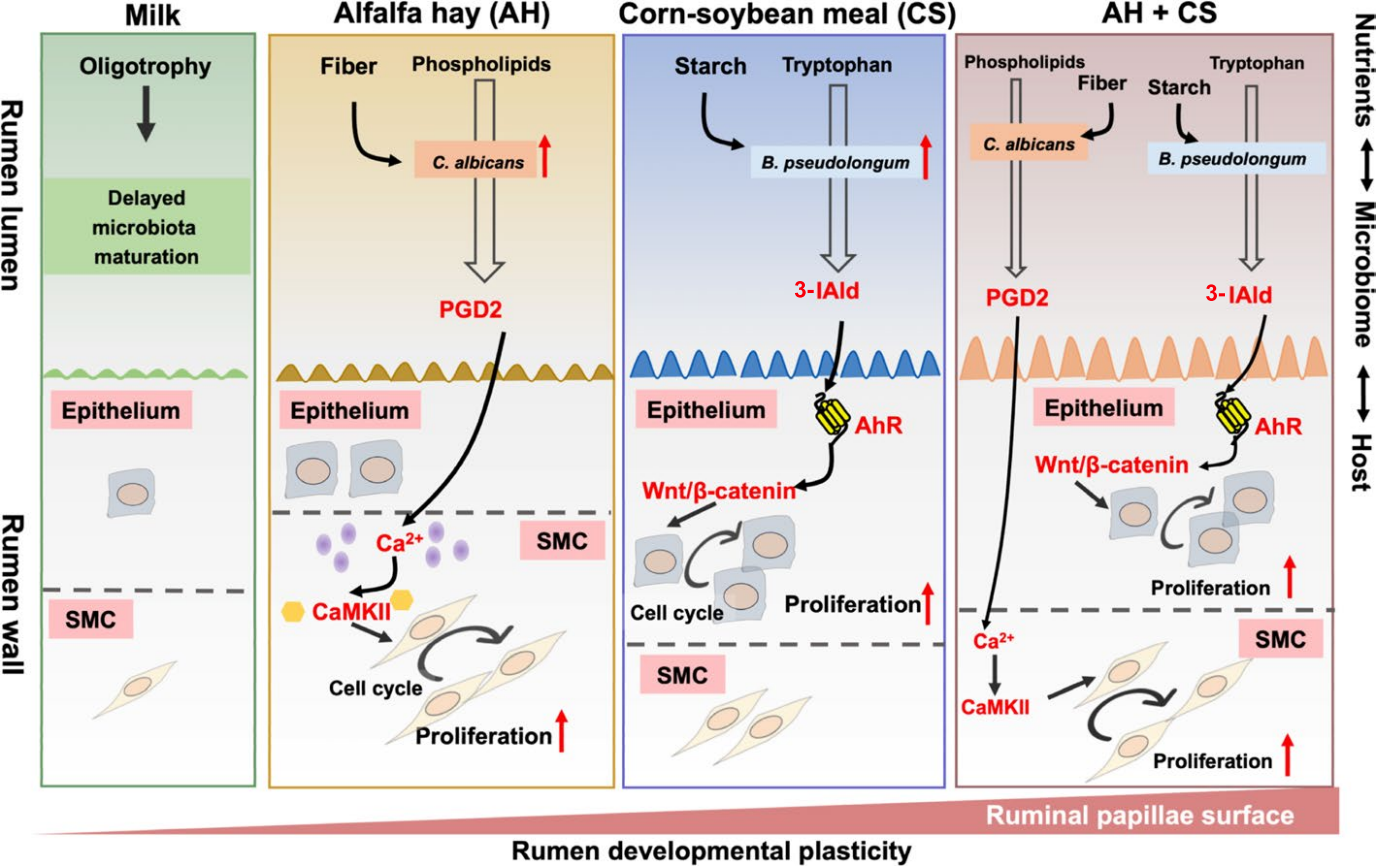
Varied dietary regimens drive early-life disparity in rumen epithelial and muscular development by microbial–host interactions. *AhR*, Aryl hydrocarbon receptor; 3-IAld, Indole-3-carboxaldehyde; PGD2, Prostaglandin D2; SMC, smooth muscle cell

Overall, the present study successfully developed a ruminal microbiome–host interactions model via massive early nutritional intervention in neonatal lambs. We found that only milk feeding delayed ruminal microbiota maturation and rumen wall development; a starchy corn-soybean starter introduction increased microbial biosynthesis of 3-IAld with the producer *Bifidobacterium pseudolongum*, which stimulated ruminal epithelial development depending on AhR-Wnt/β-catenin signaling pathway; a fiber-rich alfalfa hay diet encouraged microbial biosynthesis of PGD2 with the producer *Candida albicans*, which facilitated ruminal muscle development via the Ca^2+^ signaling pathway (Fig. 8). These findings shed new light on nutrient–microbiome–host interactions in early life. To our knowledge, this study is the first to demonstrate that early-life ruminal microbiome-derived 3-IAld and PGD2 are effective promoters of rumen development, which provide new insights into research and application of novel feed additive in young ruminants. More importantly, we also offer first direct evidence for link-specific ruminal strains and their downstream metabolites to host rumen wall physiology, which is one key to open the “black box” of rumen.

## Methods

### Solid diet introduction experiment design

The animal experiment was conducted on a sheep breeding conservation farm (Huzhou, Zhejiang Province, China) from September to November in 2018. The detailed description of animal welfare during lamb experiment is presented in Additional file 9. Forty healthy Hu lambs (11-day-old) were separated from their dams and fed mixed goat milk using a nursing bottle (water: goat milk powder = 10:1). After 3 days of adaptation, the lambs were randomly assigned to four groups, including only milk feeding (M, *n* = 10), milk plus alfalfa hay feeding (MH, *n* = 10), milk plus corn-soybean starter feeding (MC, *n* = 10), and milk plus alfalfa hay and corn-soybean starter feeding (MHC, *n* = 10). The animals were selected at a comparable initial body weight. The lambs in M group consumed mixed goat milk powder (23.85% crud protein, 25.30% fat, and 36.30% lactose) at will. The lambs in MH, MC, and MHC groups were fed 600 mL/day milk equaling 10% of their initial average body weight [50, 51] and could freely get access to solid feed. The mixed goat milk powder was provided four times daily (07:00, 12:00, 17:00, and 22:00), and the solid diets were provided twice daily (08:00 and 17:00). The nutritional levels of solid diet were shown in Additional file 10: Table S8. At 42 days of age, eight healthy lambs per group were randomly selected and stunned using electric shock. After slaughter using exsanguination, all rumen content was immediately mixed, and 10 g of ruminal content was stored in liquid nitrogen for microbial DNA extraction. The ruminal fluid sample was strained through four layers of sterile cheesecloth and stored in liquid nitrogen for the analysis of metabolome. After measuring rumen volume and weight, one portion of the rumen wall (3 × 3 cm) from the ventral sac was collected for morphology analysis using histomorphometry microscopy, as described previously [52]. After being washed three times in cold phosphate buffer saline, the ruminal epithelium and muscle layer tissue were collected and stored in liquid nitrogen for RNA extraction.

### 3-IAld and PGD2 infusion experiment design

The 3-IAld and PGD2 infusion studies were conducted on a sheep breeding conservation farm (Huzhou, Zhejiang Province, China) from February to April in 2022. The detailed description of animal welfare during lamb experiment was presented in Additional file 9. Thirty healthy male Hu lambs (11 days of age) were separated from their dams and fed mixed goat milk using an artificial nursing bottle (water: goat milk powder = 10:1). After 3 days of adaptation, 24 lambs with good adaptation to artificial nursing were randomly assigned to three groups with the following treatments: normal saline infusion (Con, *n* = 8), 3-IAld infusion (3-IAld, *n* = 8), and PGD2 infusion (PGD2, *n* = 8). There was no significant difference in initial body weight among three groups. All lambs were provided mixed goat milk powder at their will, and the feeding method was the same as M group lambs. 3-IAld (Aladdin, China) was delivered daily for 28 days by oral infusion at a dose of 30 mg/kg body weight per day in a vehicle of DMSO/saline (1:79). First, the 3-IAld powder was dissolved in vehicle at a concentration of 18.75 mg/mL; then, the 3-IAld group lambs received an oral infusion at 1.6 mL/kg body weight per day. PGD2 (Santa Cruz, CA) was delivered daily for 28 days by oral infusion at a dose of 20 µg/kg body weight per day in a vehicle of DMSO/ saline (1:79). First, the PGD2 powder was dissolved in vehicle at a concentration of 12.5 µg/mL; then, the PGD2 group lambs received an oral infusion at 1.6 mL/kg body weight per day. The Con group lambs received a vehicle at 1.6 mL/kg body weight by oral infusion. The amount of infusion was adjusted weekly according to body weight. The physiological concentrations of 3-IAld and PGD2 in the rumen liquid were 0.21 mg/mL and 0.15 µg/mL. Oral infusion dose was calculated by the physiological concentration of 3-IAld or PGD2 multiplied by the rumen volume (42 days of age), and then divided by body weight (42 days of age) of lambs. Targeted metabolomic analysis of 3-IAld was performed as previously reported [53]. The measure method of absolute concentration of PGD2 was detected by various species PGD2 ELISA kit (Cloud-clone corp., USA). During the feeding and oral infusion period, lambs with diarrhea, pneumonia, and aphtha were excluded. At 42 days of age, six healthy lambs per group were slaughtered and the ruminal epithelium and muscle layer tissue were collected for morphology analysis and RNA extraction.

### Microbial culture experiment design

*Bifidobacterium pseudocatenulatum DSM 20438, Bifidobacterium longum ATCC15707* and *Bifidobacterium adolescentis BNCC134301* were obtained from the DSMZ (Braunschweig, Germany), ATCC (Manassas, VA, USA), and BeNa Culture Collection (Beijing, China), respectively. *Bifidobacterium pseudocatenulatum DSM 20438* and *Bifidobacterium adolescentis BNCC134301* were cultured for 16 h at 37 °C in DSM medium 58 under an anaerobic workstation. *Bifidobacterium longum ATCC15707* was cultured for 16 h at 37 °C in ATCC medium 2107 under an anaerobic workstation. *Candida albicans BNCC186382* was obtained from the BeNa Culture Collection (Beijing, China), and cultured for 24 h at 37 °C in PYG medium (Beijing, China) under a constant temperature incubator.

At the initial stationary phase of strain growth, 30 mL bacterial solution was centrifuged at 4000 r at 4 °C for 20 min. The supernatant were removed and bacteria were washed using pure M9 minimal microbial growth medium (Sigma, USA). After re-centrifuged with the same condition, 15 mL M9 culture media was added to the tube and divided into three sterile 15-mL tubes (Gbico, USA). Then, the bacteria were cultured for 0, 2, 6, and 12 h under anaerobic conditions (5% CO_2_, 10% H_2_, and 85% N_2_). For the *Bifidobacterium* species, the M9 medium contained 1 mM Trp (Sigma, USA). For the *Candida albicans*, the M9 medium contained 1 mM arachidonic acid (Sigma, USA) or lecithin (Sigma, USA). Then, a 200 μL strain medium or medium control was mixed with 800 μL precooled methanol containing 1 μg/mL 4-chloro-phenylalanine (Sigma, USA) and the mixture was stored in a − 80 °C refrigerator for 30 min to remove protein. The mixture was centrifuged at 18,000 × *g* under 4 °C for 10 min to obtain supernatant. After vacuum concentration and centrifuge treatment, the final samples were obtained for UPLC-Q-TOF/MS analysis.

### Cell culture experiment design

Primary ruminal epithelia cells and smooth muscle cells were isolated from the rumen wall of Hu lambs (42 days of age), as described in an early report, with some modifications [54]. Briefly, the ventral blind sac of the rumen wall (10*10 cm) was collected and washed with ice-cold D-hanks containing 100 U/mL of penicillin and 100 mg/mL of streptomycin. Then, the ruminal epithelia and muscle layers were separated and digested repeatedly with 0.25% trypsin. Digestion with trypsin was stopped until enough individual epithelial or smooth muscle cells appeared in the digestion solution. The obtained cell suspension was centrifuged and washed with PBS. Then, the cells were cultured in F12/DMEM medium (GBICO, New York, USA) with 10% FBS (GBICO, New York, USA) and 1 × antibiotic–antimycotic (GBICO, New York, USA). The morphology and identity of primary rumen epithelial cells or rumen smooth muscular cells were authenticated using microscopic observation (Additional file 1, Fig. S9a) and immunofluorescent staining of specific protein markers (Additional file 1, Fig. S9b and c). The specific protein marker keratin 14 (KRT14) of rumen epithelial cells was stained with rabbit anti-KRT14 (1:200; Abclonal; A15069). The specific protein marker α-smooth muscle actin (αSMA) of smooth muscular cells was stained with rabbit anti-αSMA (1:800; Proteintech; 14,395–1-AP). The images were visualized with laser scanning confocal microscopy (Zeiss LSM 900/Axio Observer 7, Jena, Germany). After adherent purification, the cells underwent serum starvation (0.5% FBS) for 24 h.

To investigate the effect of 3-IAld on primary ruminal epithelia cell proliferation, the cells were treated with DMSO (control group) or 3-IAld (3-IAld group; Sigma Aldrich, Saint Louis, MO). According to previous studies [48, 55] and our pre-experimental results, we observed that 30 μM concentrations of 3-IAld had the best effect (Additional file 1: Fig. S8a). The cells were cultured for 24 h for RNA extraction and analysis of cell cycle process, cell proliferation ratio, and protein fluorescence intensity. CH223191 (MedChem Express, San Diego, USA) and XAV-939 (MedChem Express, San Diego, USA) are the specific inhibitors of AhR and β-catenin protein. The cells were pretreated with CH223191 (10 uM, 3-IAld + AhR inhibitor group) and XAV-939 (5 µM, 3-IAld + CON inhibitor group) for 1 h before addition of 3-IAld. The control and 3-IAld groups were treated with DMSO instead of the inhibitor. To identify the effect of PGD2 on primary ruminal smooth muscle cell proliferation, the cells were treated with DMSO (control group) or PGD2 (Sigma Aldrich, Saint Louis, MO; PGD2 group). According to previous studies [56, 57] and our pre-experimental results, we found that 0.10 μM concentrations of PGD2 had the best effect (Additional file 1: Fig. S8b). Cells were cultured for 24 h for RNA extraction and analysis of cell cycle process and EdU^+^ label ratio. The cells were pretreated with KN-93 (10 µM, a specific inhibitor of the CAMK2 protein; 3-IAld + CAMK2 inhibitor group) for 1 h before PGD2 addition. The control and PGD2 groups were treated with DMSO instead of the inhibitor. For all cell culture experiment, the MycoBlue Mycoplasma Detector (Vazyme, China) was used to test and eliminate mycoplasma contamination.

### Histological measurements

Rumen papilla morphology and light microscopy histomorphometric analysis were carried out through the method described by previous report [52]. In short, a piece of ruminal tissue (1 × 1 cm) was counted for the density of ruminal papillae, and then, 15 papillae were randomly selected for measuring length and width of ruminal papillae using a sliding caliper. The ruminal epithelial absorption area was calculated as papillae length × width × density × 2. Rumen wall samples were fixed in 4% paraformaldehyde and processed for paraffin imbedding and sectioning. Hematoxylin and eosin staining was used to measure the thickness of ruminal epithelia and smooth muscle layer through Image-Pro Plus 6.0 (Media Cybernetics Inc., Bethesda, MD). Three ruminal papillae per sample were selected for analysis, and five images were captured per papillae. The mean values of 15 replicate images per specimen were calculated, and different specimens were considered as repeated measures.

### Metabolomics and analysis

For GC–MS analysis, 100 μL of rumen liquid samples was mixed with 800 μL of methanol. The mixed sample was vortexed for 30 s and placed for 1 h at − 20 °C. Subsequently, the samples were centrifuged at 12,000 rpm at 4 °C for 15 min. The 200 μL supernatant was collected and then evaporated until dry at room temperature. After evaporation, the samples were derivatized by shaking them with 35 μL of methoxyamine hydrochloride (20 mg/mL) in pyridine for 90 min incubation at 37 °C. Then, the samples were trimethylsilylated by adding 35 μL of BSTFA and incubating them for 1 h at 70 °C and 1 h at room temperature. The supernatant was used for GC–MS analysis. A gas chromatography system (Agilent 6890A/ 5973C, Palo Alto, CA, USA) coupled with a Pegasus HT (LECO, Shanghai, China) time-of-flight mass spectrometer (GC-TOF–MS) was used to identify the metabolites fitted with a DB-5MS capillary column (30 m × 0.25 mm × 0.25 μm; J&W Scientific, Folsom, CA, USA). Here, 1 μL aliquot of the sample was injected in splitless mode. Helium was used as the carrier gas. The primary temperature was kept at 70 °C for 2 min before being increased to 200 °C at a rate of 10 °C /min and then raised to 280 °C at a rate of 5 °C/ min and kept at this temperature for 6 min. The column effluent was fully scanned in the mass range 50–550 m/z. The data was performed feature extraction and preprocessed with XCMS in R software, and then normalized and edited into two-dimensional data matrix by excel 2016 software, including retention time, mass-to-charge ratio, and observations and peak intensity. Finally, using NIST library identify matching metabolites though retention time and m/z.

For UPLC-Q-TOF/MS analysis, 20 μL of rumen liquid samples was mixed with 200 μL of methanol. The mixed sample was vortexed for 30 s and placed for 1 h at − 20 °C. Subsequently, the samples were centrifuged at 18,000 rpm and 4 °C for 15 min. The 100 μL supernatant was collected and then evaporated until dry at room temperature. The sample was reconstituted in 100 μL of methanol (LC–MS grade, Merck) before analysis on an LC − MS/MS. For less polar compounds, LC separation was conducted on an Atlantis T3 (100 mm × 2.1 mm, 3.0 μm; Waters) using a gradient of solve A (5 m Mammonium formate and 0.05% formic acid buffer) and solvent B (acetonitrile). Next, 5 μL of the sample was injected. The flow rate was 0.25 mL/ min. The gradient was 0–3 min, 5% B; 3–8 min, 5–65% B; 8–10 min, 65–95% B; 10–12.5 min, 95% B; 12.5–13 min, 95–5% B; 13–17 min, 5% B. For polar compounds, LC separation was conducted on a XBridge BEH Amide column (4.6 mm × 100 mm, 3.5 μm; Waters) using a gradient of solve A (15 mM ammonium acetate and 0.3% ammonia buffer, pH = 9) and solvent B (acetonitrile). After this, 5 μL of the sample was injected, and the flow rate was 0.4 mL/ min. The gradient was 0–1 min, 85% B; 1–12 min, 85–30% B; 12–13 min, 30% B; 13–14 min, 30–85% B; 14–27 min, 85% B. The 6545 XTQ-TOF mass spectrometer was operated with a spray voltage of − 3.5 kV in negative mode and + 4 kV in positive mode. Drying gas was set at 9 L/min, and sheath gas was set at 110 L/min. The sheath gas temperature was 325 °C. Fast data-dependent acquisition (DDA) MS/MS experiments were performed with a collision energy map in the mass range 50–1000 m/z. The Progenesis QI (Nonlinear Dynamics, Newcastle, UK) was used for peak picking and alignment. Molecular identification of the assigned biomarkers was accomplished by matching the acquired precursors and fragment ions against several standard metabolome databases, including the Human Metabolome Database (http://www.hmdb.ca/), MassBank (http://www.massbank.jp/index.html), and METLIN (http://metlin.scripps.edu/index.php). Partial metabolite identification was further confirmed by comparison with the available standards.

The web-based tool Metabo Analyst 6.0 (https://www.metaboanalyst.ca/) was used for data normalization and analysis, along with for metabolites functional enrichment. The peak area data was normalized by sample median, log transformation and autoscaling. The differential metabolites were determined by a fold-change threshold of 2 and false discovery rate (FDR) of < 0.05 from the Wilcoxon rank-sum test and Benjamini and Hochberg multiple testing correction.

### Clustering of ruminal metabolites via WGCNA analysis

R software package WGCNA 1.69 [58] was used to identify key phenotype-related metabolic modules based on correlation patterns. The Pearson correlation matrix was calculated for all possible metabolite pairs and then transformed into an adjacency matrix with soft thresholding power set to 8 for the best topological overlap matrix. A dynamic tree cut algorithm was used to detect groups of highly correlated metabolites. The minimum module size was set to 5, and the threshold for merging module was set to 0.25 as default. The profile of each metabolite cluster was summarized by the mainly class of metabolites. Each module was assigned a unique color and contained a unique set of metabolites. These resulting modules containing metabolites highly correlated with one another were then used in data integration to identify the relationships between rumen metabolites and rumen development phenotypes.

### Generation of random forests classification models

In the present study, the random forest was used to select differentiating biomarkers among the different groups. A random forest classification was performed using the Random Forest package in R (version 3.6.2) with 500 trees and tenfold cross-validation to obtain robust estimates of the generalization error and feature importance. We used out-of-bag (OOB) error rate to measure the performance of the model. Our dataset was partitioned into a training set (including 70% of the samples) and a validation set (including the remaining 30%). Prism 8 (GraphPad Software, La Jolla, CA, USA) was used to construct a graph of TOP 20 metabolites based on mean decrease accuracy value.

### Shotgun metagenome sequencing and analysis

Genomic DNA of the rumen content microbiota was extracted using a DNA Kit (EZNA, Omega Bio-Tek, Norcross, GA) according to the manufacturer’s protocols. The quantity and quality of the microbial DNA were examined using a NanoDrop 2000 spectrophotometer (Thermo Fisher Scientific) and 1.0% agarose gel electrophoresis. All extracted DNA samples were stored at − 80 °C until subsequent processing. Genomic DNA was used with Illumina’s TruSeq for library preparation. Libraries were pooled, and paired-end sequencing was conducted on an Illumina HiSeq PE 150 Platform. Then, BWA (version 0.7.12) [59] and Fast QC (version 0.11.8) [60] were utilized to delete the adaptors, low-quality reads, and *ovis aries* and diet (maize, medicago, soybean, and wheat) contaminations in the sequencing raw data. The obtained clean reads were assembled using MEGAHIT (version 1.1.1) [61] based on the option of min-contig-len 500. We used Prodigal (version 2.6.3) [62] to predict the gene function depending on contigs from each sample and took advantage of CD-HIT to cluster the assembled contigs depending on the 95% cutoff sequencing identity. Finally, the pan-metagenome was used to analyze changes in metagenome functions. Entries in all the gene catalogs were subjected to taxonomic and functional assignment using DIAMOND [63] (v.0.9.22) based on BLASTP searches against the NCBI-NR (October 2018; approximately 550 M sequences) and KEGG [64] (v.90.0) databases (parameter: –evalue 0.00001 –max-target-seqs 10). The high-quality reads from each sample were aligned against the gene catalogs using BWA-MEM [59] (v.0.7.17), and abundance profiles of genes (alignment length ≥ 50 bp and sequence identity > 95%) were calculated in transcripts per million (TPM) [65], with corrections for variations in gene length and mapped reads per sample. TPM is calculated as:

$$TPM=\frac{N{\text{g}}}{L{\text{g}}}\times \frac{1}{\sum {\text{j}}\frac{N{\text{j}}}{L{\text{j}}}}\times 10$$

where *N*_*g*_ is the read count, i.e., the average number of reads mapped to the *g* gene; and *L*_*g*_ is the gene length, i.e., the number of nucleotides in the *g* gene. The index *j* stands for the set of all genes determined in a catalog, and *g* is an index indicating a particular gene [65]. The relative abundances of taxa and KOs were calculated from the abundances of annotated genes [66]. Briefly, for the taxonomic profiles, we used phylogenetic assignment of each annotated gene from the rumen microbial gene catalog and summed the relative abundances of genes from the same phylum or genus to produce the abundance of each phylum or genus. The profile of each KO was calculated using the same process. The relative abundance of a KEGG pathway was calculated from the summation of the relative abundances of its contained KOs. The parameters or codes of software and packages mentioned were descripted in the previous studies [46, 67].

### Transcriptome analysis

Total RNA was extracted by TRIzol (Invitrogen Life Technologies, Carlsbad, CA, USA), and concentrations and purity were measured using NanoDrop (NanoDrop Technologies, Wilmington, DE, USA). An RNA Nano 6000 Assay Kit was used to assess integrity (Agilent Technologies, CA, USA). Here, 1000 ng RNA per sample was used as the input material for the cDNA library construction. Sequencing libraries were generated by the NEBNextUltraTM RNA Library Prep Kit for Illumina (NEB, USA) according to the manufacturer’s instructions. To select cDNA fragments that were preferentially 240 bp in length, the library fragments were purified with the AMPure XP system (Beckman Coulter, Beverly, USA) and then amplified by PCR. Finally, PCR products were purified (AMPure XP system), and library quality was assessed using the Agilent Bioanalyzer 2100 system. The library preparations were sequenced on an Illumina platform, and paired-end reads were generated.

Clean reads were processed by deleting low-quality reads, reads with adaptor sequences, and reads including 0.5% unknown bases in raw reads, which were then mapped to the *ovisaries* reference genome (Oar v3.1) using TopHat (http://tophat.cbcb.umd.edu/; v2.0.9). Gene expression levels were calculated by fragments per kilobase of transcript per million fragments mapped (FPKM). The DEGs changed by diet were identified by comparing any two groups using the DESeq R package (1.10.1). The DEGs were identified by the FDR < 0.05 based on Benjamini and Hochberg multiple testing correction, as well as a fold-change (FC) of > 1.5 or < 0.667. Then, genes that were differentially expressed in any two groups were identified as DEGs. Finally, the GO enrichment analysis of DEGs was carried out by DAVID (version 6.8). KOBAS (version 3.0) was used to test the statistical enrichment of DEGs in the KEGG pathways.

### Cell proliferation, protein fluorescence intensity, and quantitative real-time PCR

Cell proliferation was detected by incorporating EdU according to the EdU 594 cell proliferation kit (Beyotime, Shanghai, China), following the manufacturer’s instructions. The process of cell cycle analysis was according to the previous description by Gui et al. [54]. The cell immunofluorescence assay was performed as the following methods. The cells were fixed with paraformaldehyde for 30 min, permeabilized with 0.3% Triton X-100 for 15 min, incubated in 5% BSA for 1 h, and then incubated with primary antibodies (anti-rabbit AhR antibody, 1:200; anti-rabbit CYP1A1 antibody, 1:100; anti-rabbit β-catenin, 1:200; anti-rabbit CAMK2, 1:200; Proteintech) overnight at 4 °C. Next, the cells were incubated with a goat anti-rabbit antibody conjugated to Alexa Fluor 594 (1:500, Abcam) for 60 min at room temperature. Nuclei were stained with DAPI (1:5000, Invitrogen) for 5 min. The samples were examined with a Zeiss 710 laser scanning confocal microscope. Fluorescence images were collected for further qualitative and quantitative analyses.

The total RNA extract, cDNA synthesis, and real-time quantitative PCR were performed according to Lin et al. [46]. Next, qRT-PCR of all genes was performed using the QuantStudio 7 flex Real-time PCR Instrument (Applied Biosystems, Foster City, CA, USA) with fluorescence detection of SYBR green dye. Amplification conditions were as follows: 30 s at 95 °C followed by 40 cycles composed of 5 s at 95 °C, 34 s at 60 °C, 15 s at 95 °C, 60 s at 60 °C, and 15 s at 95 °C. Glyceraldehyde 3-phosphate dehydrogenase was used as a housekeeping gene to normalize the mRNA levels of each gene. The primers and amplicon sizes of all genes are presented in Additional file 10: Table S9.

### Statistical analysis

SPSS version 22.0 (SPSS, Inc) was used for statistical analysis unless otherwise indicated. For body weight and daily nutrient intake of lambs, the statistical analysis was performed using the mixed linear model. The treatment, age, and their interaction were treated as fixed factors and the lamb was considered as a random effect. When significant differences were found among different treatment, a multiple comparison of body weight at 42 days of age was conducted based on one-way ANOVA. Rumen organ index and qRT-PCR results in vivo experiment were tested using one-way ANOVA followed by post hoc Tukey tests in SPSS software. Rumen papillae morphology and rumen wall thickness were measured using the mixed effects models (MIXED) procedure of SPSS, with diet as the fixed effect and lamb as the random effect. The data of microbial incubation was analysis by independent sample *t*-test, while the results of temporal variation was tested using one-way ANOVA followed by post hoc Tukey tests. For data of cell culture experiment, an independent sample *t*-test in SPSS software packages was used to assess statistical significance. A value of *P* < 0.05 was considered statistically significant. PCA (“vegan” package) and correlation analysis (“ggpubr” package) were conducted using related packages in R (version 3.6.2). Using G*Power (3.1.9.6), we performed a post hoc analysis to determine the effect size for animal experiment and our samples could detect with adequate power (80%) based on an alpha = 0.05, and *F*-test using one-way ANOVA.

### Supplementary Information

**Additional file 1: Supplementary Figures.** This additional file contains the supplementary figures (Fig. S1-S9).

**Additional file 2: Table S1.** The ruminal metabolites involved in the M6 and M9 module via weighted gene co-expression network analysis.

**Additional file 3: Table S2.** The Gene Ontology terms of differentially expressed genes in the ruminal epithelia transcriptome.

**Additional file 4: Table S3.** The Gene Ontology terms associated with rumen epithelial development biological process.

**Additional file 5: Table S4.** Correlation analysis (FDR < 0.10) of ruminal epithelial thickness and gene expression related to the Wnt/β-catenin signaling pathway.

**Additional file 6: Table S5.** The Gene Ontology terms of differentially expressed genes in the ruminal muscle transcriptome.

**Additional file 7: Table S6.** The Gene Ontology terms associated with rumen muscle development biological process.

**Additional file 8: Table S7.** Correlation analysis (FDR < 0.10) of ruminal muscle layer thickness and gene expression related to the Ca^2+^ signaling pathway.

**Additional file 9.** Animal welfare.

**Additional file 10: Table S8 and S9.** Table S8, ingredient and chemical composition of alfalfa hay and corn-soybean starter; Table S9, primers for quantitative real-time PCR.

**Additional file 11.** The metabolic profiling data generated in this study.

**Additional file 12. **Review history.

## Data Availability

The metabolomic datasets analyzed in this study have been deposited in the OMIX database of China National Center for Bioinformation/Beijing Institute of Genomics, Chinese Academy of Sciences under accession number OMIX005843 (https://ngdc.cncb.ac.cn/omix/release/OMIX005843) [68]. The metagenomic datasets analyzed in this study have been deposited in the Genome Sequence Archive database of China National Center for Bioinformation/Beijing Institute of Genomics, Chinese Academy of Sciences  under accession number CRA014962 (https://ngdc.cncb.ac.cn/gsa/browse/CRA014962) [69]. The transcriptomic datasets analyzed in this study are available at National Center for Biotechnology Information under accession numbers PRJNA881929 (https://www.ncbi.nlm.nih.gov/bioproject/PRJNA881929) [70]. All codes in this paper are publicly available at GitHub (https://github.com/sundm128/Genome-2023) [71],  and at Zenodo (DOI: 10.5281/zenodo.10668958) [72]. The source code is released under the MIT license. The metabolic profiling data generated in this study are presented in Additional file 11.
