# Supplementary material for: Early-life ruminal microbiome-derived indole-3-carboxaldehyde and prostaglandin D2 are effective promoters of rumen development
Source: Genome Biol. 2024 Mar 4;25:64. doi: 10.1186/s13059-024-03205-x (PMC10910749; doi:10.1186/s13059-024-03205-x)
Supplement: Supplementary file 1 — Additional file 1: Supplementary Figures. This additional file contains the supplementary figures (Fig. S1-S9). [file 13059_2024_3205_MOESM1_ESM.docx]

Fig. S1.

**Fig. S1.** The heatmap of the top 50 significantly (*FDR* < 0.05) different metabolites among groups, and the difference among the four groups was identified using the Kruskal-Wallis test (n = 8 per group). MH: milk plus alfalfa hay, MC: milk plus corn-soybean starter, MHC: milk plus alfalfa hay and corn-soybean starter.

Fig. S2.


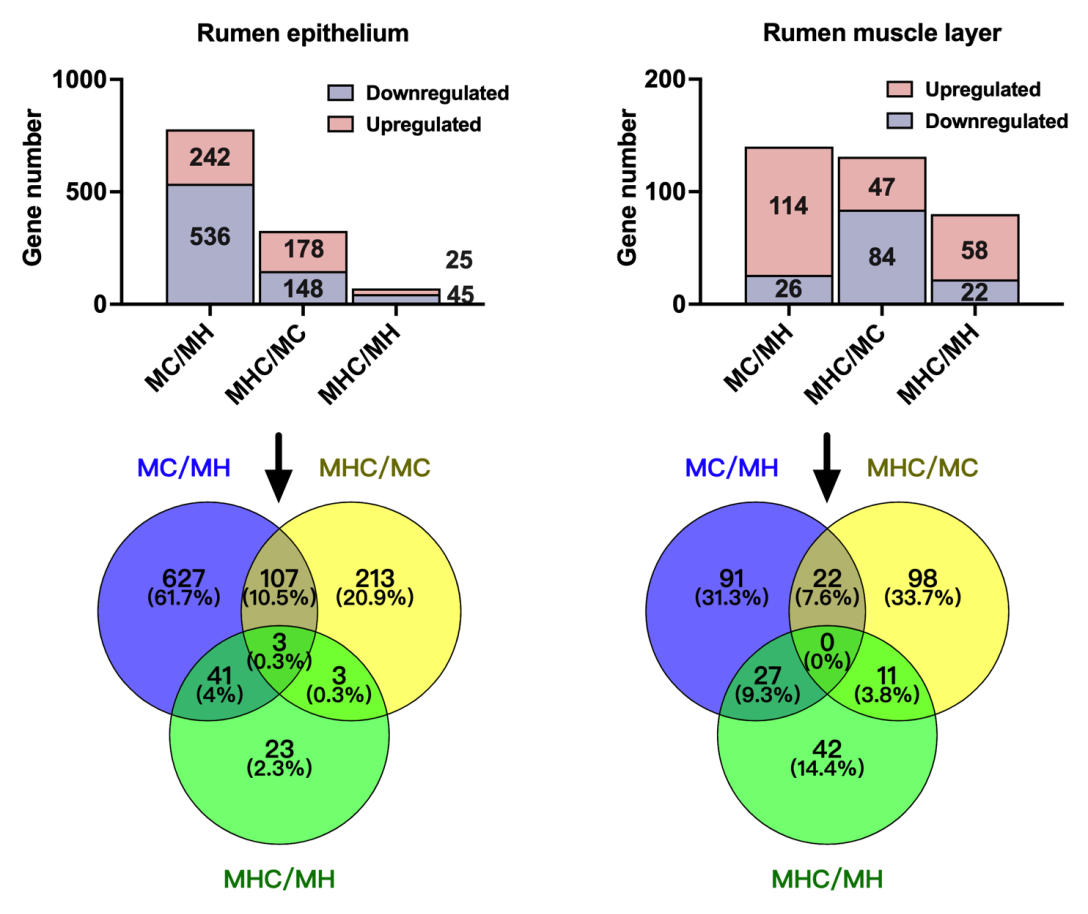


**Fig. S2**. The number of differential expressed genes in the ruminal epithelia and muscle layer (FC>1.5 and FDR<0.05). MH: milk plus alfalfa hay, MC: milk plus corn-soybean starter, MHC: milk plus alfalfa hay and corn-soybean starter.

**Fig. S3**.

**
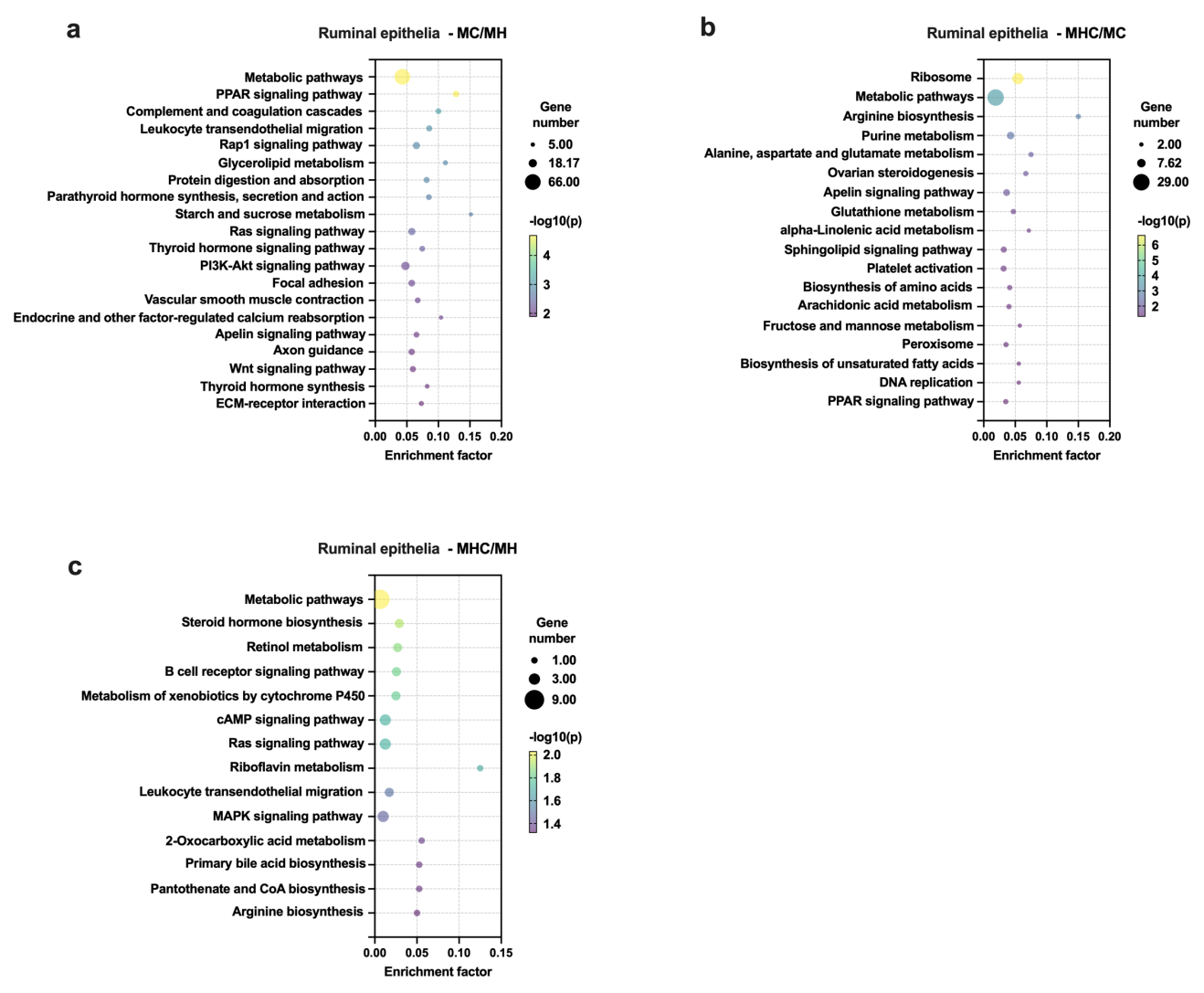
**

**Fig. S3**. KEGG enrichment pathway of differential expressed genes in each comparison based on the rumen epithelial transcriptome. MH: milk plus alfalfa hay, MC: milk plus corn-soybean starter, MHC: milk plus alfalfa hay and corn-soybean starter.

**Fig. S4**.

**
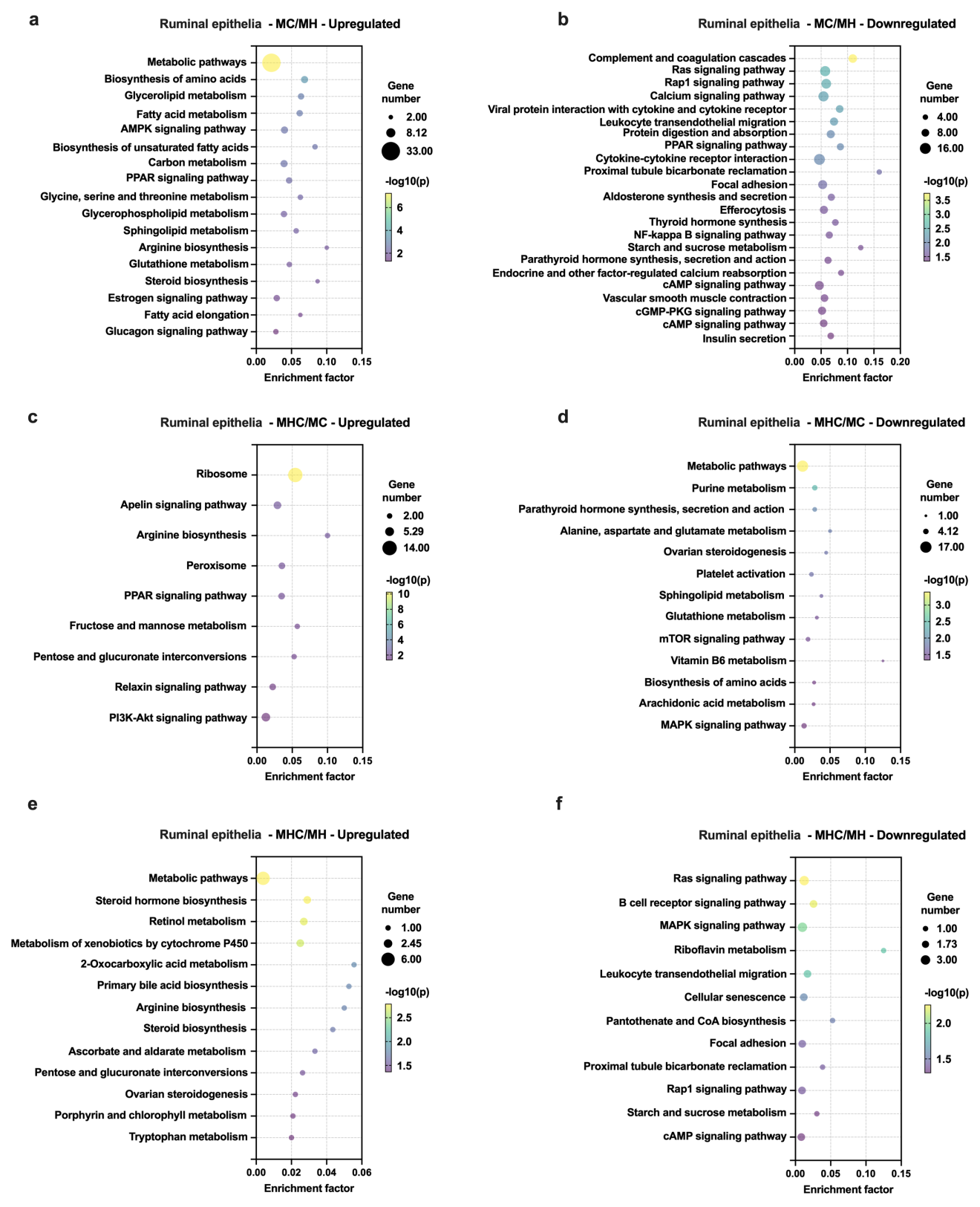
**

**Fig. S4**. The functional KEGG pathways enrichment in upregulated and downregulated genes for each pairwise comparison between MC, MH, and MHC based the rumen epithelial transcriptome. MH: milk plus alfalfa hay, MC: milk plus corn-soybean starter, MHC: milk plus alfalfa hay and corn-soybean starter.

**Fig. S5**.

**
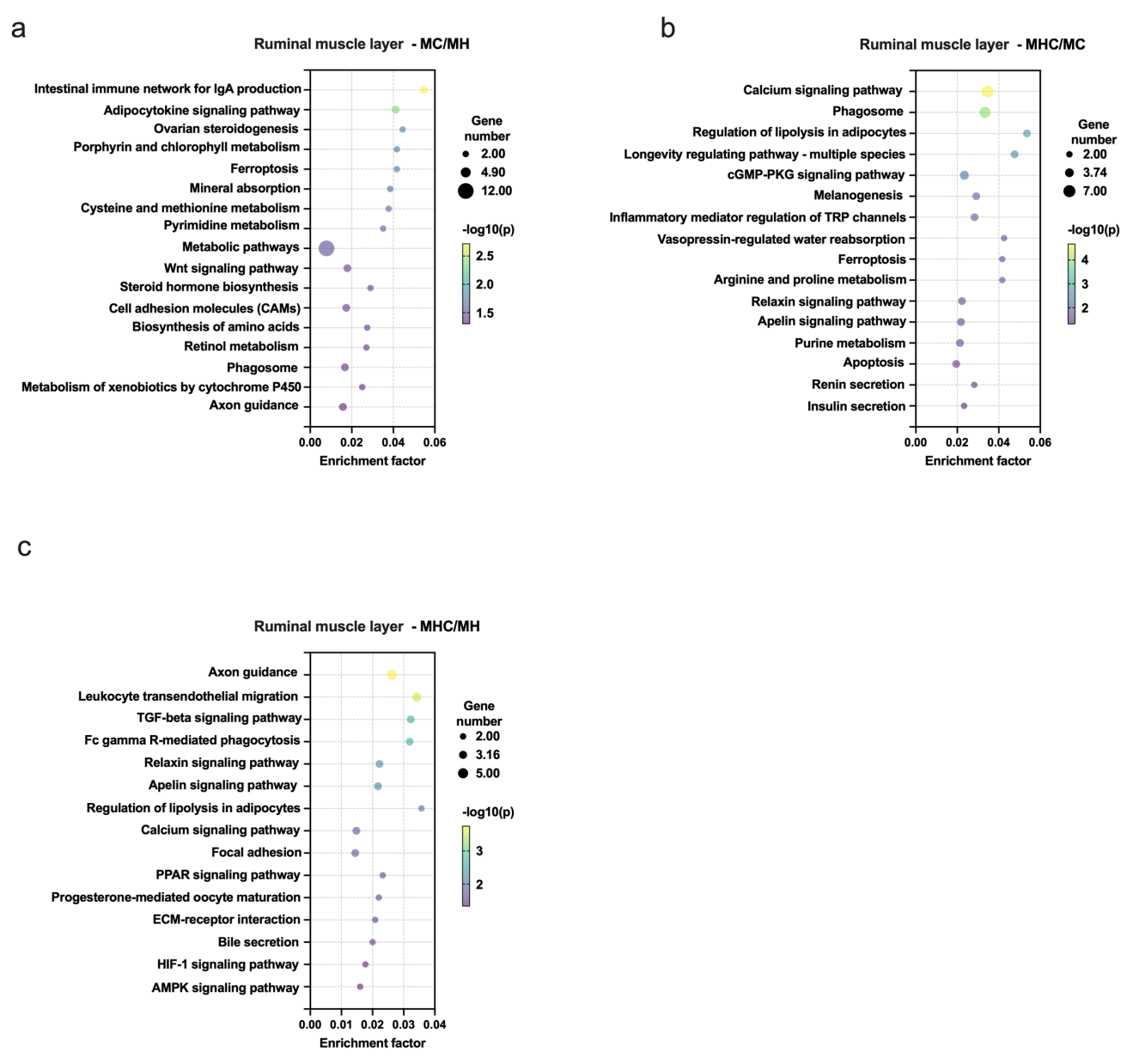
**

**Fig. S5**. KEGG enrichment pathway of differential expressed genes in each comparison based on the ruminal muscle transcriptome. MH: milk plus alfalfa hay, MC: milk plus corn-soybean starter, MHC: milk plus alfalfa hay and corn-soybean starter.

**Fig. S6**.

**
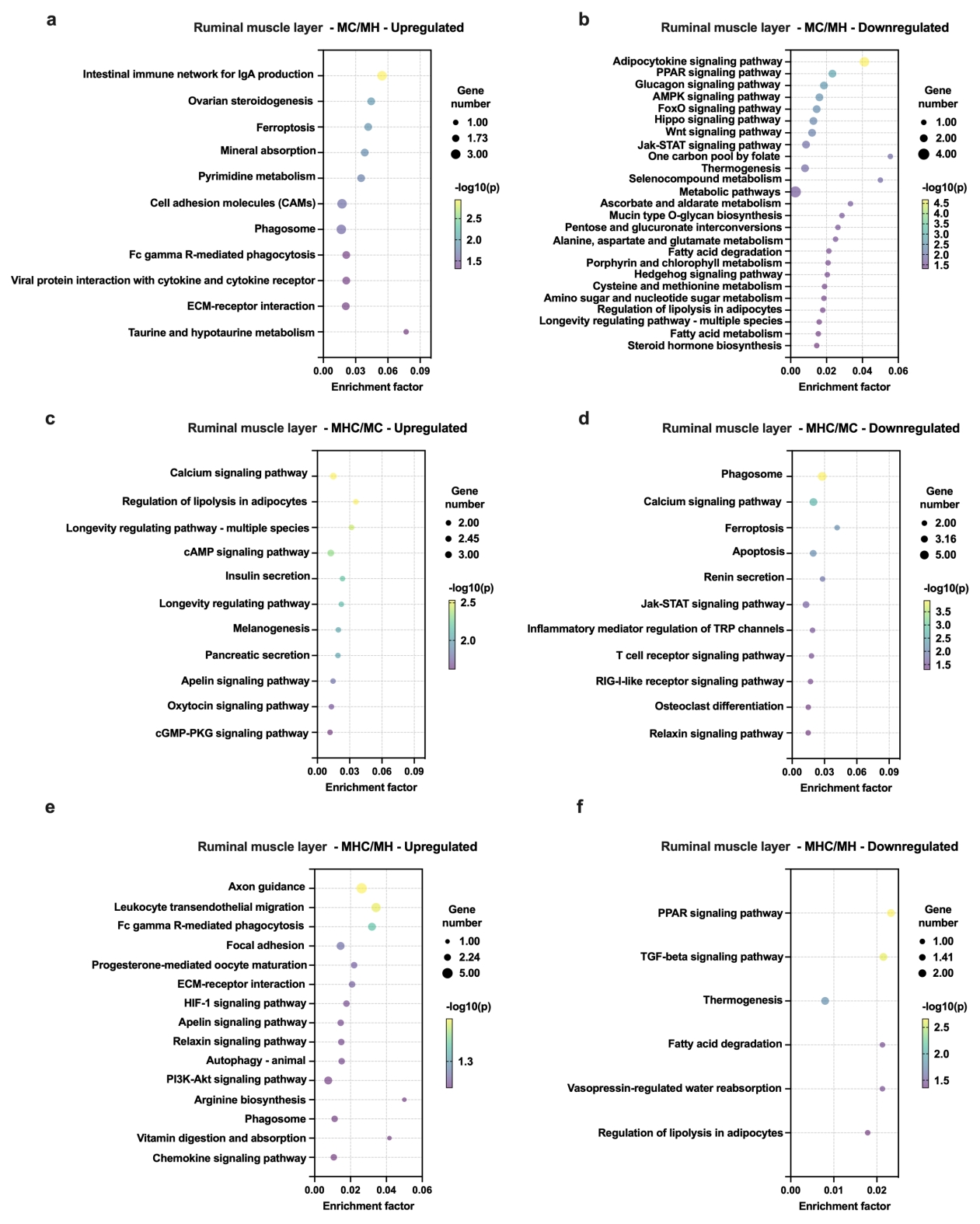
**

**Fig. S6**. The functional KEGG pathways enrichment in upregulated and downregulated genes for each pairwise comparison between MC, MH, and MHC based on the ruminal muscle transcriptome. MH: milk plus alfalfa hay, MC: milk plus corn-soybean starter, MHC: milk plus alfalfa hay and corn-soybean starter.

Fig. S7.

**Fig. S7. a,b** IAld (a) and PGD2 (b) changed the mRNA expression of the cell cycle proteins of primary rumen epithelia cells and rumen smooth muscle cells *in vitro*, respectively, n = 5. **c,d** Effect of IAld and PGD2 infusion on the relative mRNA expression of cyclins (CCNs) in the rumen epithelia (c) and muscle (d), n = 6. Data represent the mean ± SEM. Means with different letters were significantly different (*P*< 0.05) via one-way ANOVA followed by post hoc Tukey tests (c and d). **P*< 0.05 compared with the control group; # *P*< 0.05 compared with the IAld or PGD2 group via independent sample t-test.

Fig. S8.

**Fig. S8.** Pre-experimental results of primary rumen epithelia cells and primary rumen smooth muscle cells in various concentration of IAld and PGD2 in cell culture, n = 5. **a** *In vitro* 5-Ethynyl-2′-deoxyuridine^+^ (EdU^+^) assay shows that 30 μM concentration of IAld is most suitable to promote primary rumen epithelia cell proliferation. **b** *In vitro* EdU^+^ assay shows that 0.1 μM concentration of PGD2 is most suitable to promote primary rumen smooth muscle cell proliferation. Turkey’s multiple range test in one-way analysis of variance was performed to assess statistical significance. Mean values within a column with unlike superscript letters were significantly different (*P* < 0.05).

Fig. S9.


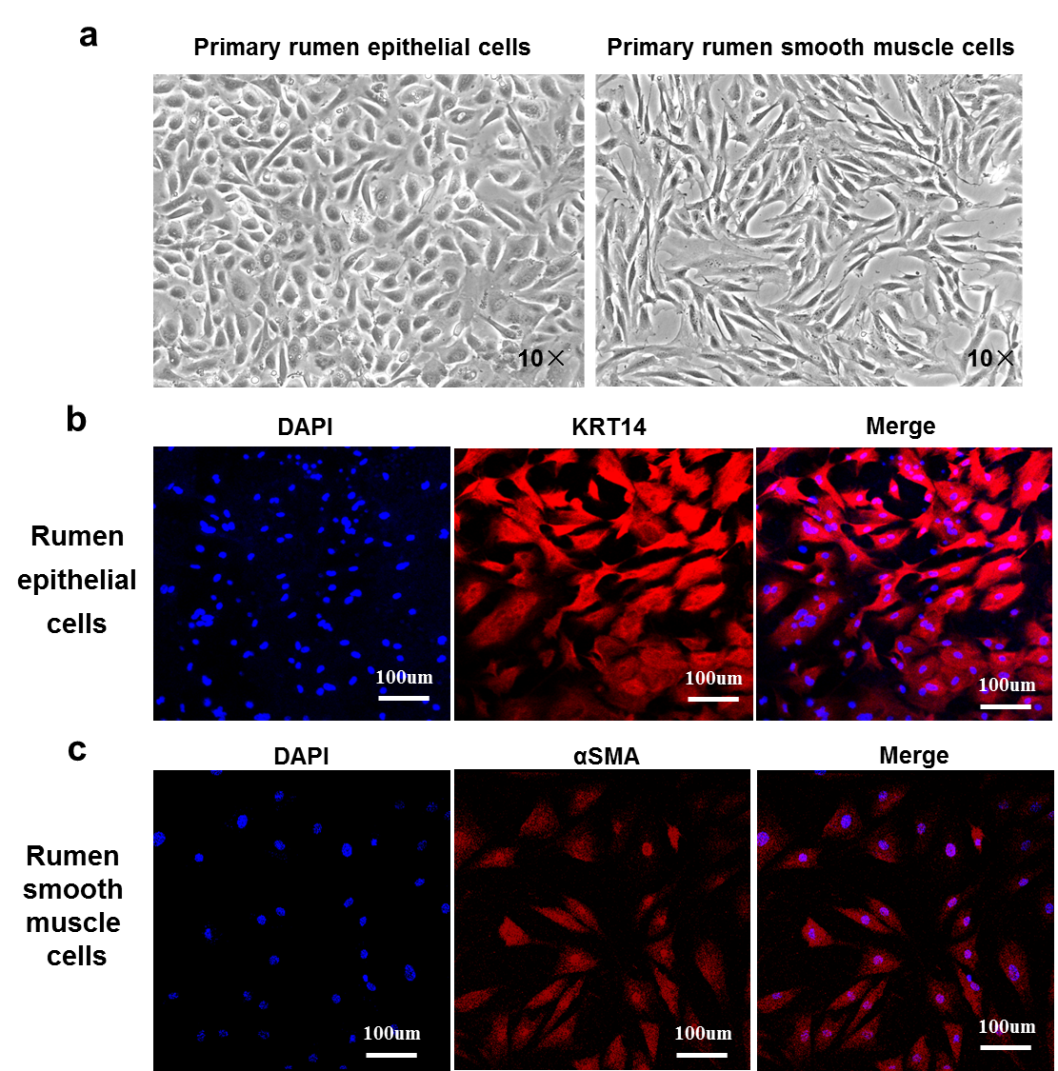


**Fig. S9.** The morphology and identity of primary rumen epithelial cells or rumen smooth muscular cells were authenticated using microscopic observation and specific molecular marker. **a** Observation of cell morphology using inverted light microscope. **b** Expression of keratin 14 (KRT14) in the primary epithelial cells; Immunofluorescence staining with antibody against KRT14, nuclei stained with DAPI, observation with laser scanning confocal microscopy. **c** Expression of α-smooth muscle actin (αSMA) in the primary smooth muscular cells; Immunofluorescence staining with antibody against αSMA, nuclei stained with DAPI, observation with laser scanning confocal microscopy.
