# Supplementary material for: Early-life ruminal microbiome-derived indole-3-carboxaldehyde and prostaglandin D2 are effective promoters of rumen development
Source: Genome Biol. 2024 Mar 4;25:64. doi: 10.1186/s13059-024-03205-x (PMC10910749; doi:10.1186/s13059-024-03205-x)
Supplement: Supplementary file 9 — Additional file 9. Animal welfare. [file 13059_2024_3205_MOESM9_ESM.docx]

**1. Animal management**

(1) The experimental personnel, who underwent training in animal welfare and feeding technology, have specialized knowledge and managerial skills in lamb feeding.

(2) These individuals exhibited a strong sense of responsibility and possessed a comprehensive understanding of the biological characteristics and behavioral patterns of lambs. They were proficient in fundamental knowledge pertaining to animal health and welfare, and possessed the ability to apply this knowledge effectively in practical settings, promptly identifying and addressing any abnormal behaviors that may arise.

**2. Feeding environment**

(1) The establishment of the lamb farm adheres to the guidelines set forth in China Technical specifications for livestock and poultry farm area design NY/T 682, as well as the stipulations regarding animal welfare.

(2) The materials utilized in the lamb house facilities and equipment were non-toxic and pose no harm, while also possessing thermal insulation capabilities.

(3) Regular inspections of the lamb housing facilities were conducted to prevent any potential harm to the sheep.

(4) It is essential to maintain a tranquil environment within the lamb house, refraining from disturbing the lambs during their resting periods.

(5) Sufficient space, exceeding 1 m^2^ per lamb, was provided to enable the lambs to move and rest freely, thereby fulfilling their welfare requirements for unrestricted movement.

(6) The experiment was conducted in winter and heating equipment was provided for the lambs to ensure that the ambient temperature was between 18 and 25 ℃.

(7) Natural lighting was used in the lamb house. Lighting equipment was also installed to meet the lamb’s need to drink milk at night

**3. Feeding management**

(1) Alfalfa hay, solid diet and feed additives complied with the requirements of Guidelines for the use of livestock and poultry feed and feed additives in China (NY/T 5032).

(2) Kept feeding equipment clean and remove remaining feed in a timely manner.

(3) To achieve a nutritional composition similar to breast milk, goat milk powder was employed for nourishment. Based on breastfeeding patterns, it was recommended to administer feedings four times daily. During each feeding, it was advised to utilize boiling water at a temperature of 60°C to prepare the milk powder, subsequently cooling it to 40°C prior to consumption.

(4) Lambs were fed by bottles with nipples. Rinse the bottle with boiling water after each use to prevent bacterial growth.

(5) Sufficient, clean and fresh drinking water were provided. The water quality met the requirements of drinking water quality standards for livestock and poultry in China (NY 5027) standards.

(6) Fresh solid diets were provided twice daily (08:00 and 17:00) to ensure that lambs had solid diets available at any time

**4. Health management**

(1) The health status of the lambs was checked every day.

(2) Prompt isolation of sick or injured lambs is essential, followed by the provision of effective treatment, attentive care, continuous observation, and meticulous feeding. Detailed records were maintained, and a minimum of two daily checks were conducted.

**5. Lamb slaughter**

(1) Slaughter managers were trained in animal welfare knowledge.

(2) The lambs were stunned using electric shock for reducing lambs suffering.

(3) The slaughtering of lambs was conducted exclusively within a professional slaughterhouse to prevent exposure to the sight of their companions being killed.
