# Supplementary material for: Early-life ruminal microbiome-derived indole-3-carboxaldehyde and prostaglandin D2 are effective promoters of rumen development
Source: Genome Biol. 2024 Mar 4;25:64. doi: 10.1186/s13059-024-03205-x (PMC10910749; doi:10.1186/s13059-024-03205-x)
Supplement: Supplementary file 10 — Additional file 10: Table S8 and S9. Table S8, ingredient and chemical composition of alfalfa hay and corn-soybean starter; Table S9, primers for quantitative real-time PCR. [file 13059_2024_3205_MOESM10_ESM.docx]

Table S8. Ingredient and chemical composition of the alfalfa hay and corn-soybean starter (dry matter basis)

| Item | Alfalfa hay | Corn-soybean starter |
| --- | --- | --- |
| **Ingredients, % DM** |  |  |
| Alfalfa hay | 98.50 | 0.00 |
| Corn | 0.00 | 56.00 |
| Soybean meal | 0.00 | 31.00 |
| Wheat | 0.00 | 4.00 |
| Whey powder | 0.00 | 5.00 |
| limestone meal powder | 0.00 | 1.00 |
| Calcium monophosphate | 0.00 | 1.50 |
| Nacl | 0.50 | 0.50 |
| Premix^1^ | 1.00 | 1.00 |
| **Nutrient composition** |  |  |
| Metabolic energy, MJ/kg DM^2^ | 9.69 | 12.67 |
| Crude protein, % DM | 18.76 | 19.62 |
| Ether extract, % DM | 2.26 | 3.68 |
| Crude fiber, % DM | 22.29 | 3.35 |
| Crude ash, % DM | 7.46 | 3.16 |
| Ca, % DM | 1.38 | 1.04 |
| P, % DM | 0.50 | 0.50 |

^1^ Contained 102g·kg-1 of Zn，47g·kg-1 of Mn，26g· kg-1 of Cu，1140 mg·kg-1 of I，500 mg·kg-1 of Se，340 mg·kg-1 of Co，17167380IU·kg-1 of vitamin A，858370IU·kg-1 of vitamin D，and 23605IU·kg-1 of vitamin E.

^2^ Calculated based on Ministry of Agriculture of China recommendations (MOA, 2004). DM, dry matter.

Table S9. Primers for quantitative real-time PCR

| Gene Name | Gene ID | Primer sequence (5’→3’) | Amplicon Size, bp |
| --- | --- | --- | --- |
| CCNA | NC_019478.2 | F: CTCTCCTATCACCGCCTGAC  R: CTTTGGGGTCCAAGTTCTGC | 144 |
| CCND1 | NC_019478.2 | F: CCTGCCGTCCATGCGGAA  R: GAACTTCACATCTGTGGCAC | 403 |
| CCNE | XM_015100542 | F: TGGCACCGATGTCTCTGTTC  R: CCACACTGGCTTCTCACAGT | 114 |
| AhR | XM_004007775.5 | F: TACGCTGGGGCTGTTTCAAT  R: AAACCAAGGTGAGGAGTGGG | 273 |
| CYP1A1 | XM_042234960.1 | F: CCAGCTGACTTCATCCCTGTC  R: AAGGCCTCCAAATAGGGCAG | 417 |
| CTNNB1 | NM_001308590.1 | F: TCACTAAGCAGTGTGTGAGGG  R: GAAGGAATTCCCCGCTCCAA | 568 |
| CASQ1 | XM_004002663.5 | F: TCCGTGGCCCAAGATAACAC  R: TTCCATCCATACGCTGTCCG | 168 |
| CAMK2A | XM_004008972.5 | F: GTCCAGTTCCAGCGTTCAGTT  R: TTCCGGGACCACAGGTTTTC | 258 |
| GAPDH | NM_001190390.1 | F: GGGTCATCATCTCTGCACCT  R: GGTCATAAGTCCCTCCACGA | 180 |
| SLC4A4 | XM_042251504.1 | F: ACCACGGCAAGAAAGCTCAT  R: GGTATGGCAGCTGCAAGGTA | 200 |
| HMCN1 | XM_004013873.5 | F: AAGCATAAAGAGTGGCCCCC  R: TATTCGCCTGCGATCTGTCC | 250 |
| KCNN2 | XM_042251962.1 | F:GCGGGGAAAACATGAAAGAGT  R:CCTTCTGCGGAACATGAGGG | 326 |
