# Supplementary material for: Early-life ruminal microbiome-derived indole-3-carboxaldehyde and prostaglandin D2 are effective promoters of rumen development
Source: Genome Biol. 2024 Mar 4;25:64. doi: 10.1186/s13059-024-03205-x (PMC10910749; doi:10.1186/s13059-024-03205-x)
Supplement: Supplementary file 12 — Additional file 12. Review history. [file 13059_2024_3205_MOESM12_ESM.docx]

Review History

**First round of review**

**Reviewer 1**

**Were you able to assess all statistics in the manuscript, including the appropriateness of statistical tests used?**

Yes, and I have assessed the statistics in my report.

**Comments to author:**

Sun et al. studied the effects of different diet on lamb ruminal development and identified two microbe-derived metabolites, IAld and PGD2, which can promote rumen epithelial and muscular development, respectively. They used metabolomics to identify IAld and PGD2 as the top metabolites to be associated with ruminal development, leveraged metagenomics to link IAld production with Bifidobacterium and PGD2 production with Candida albicans, and utilized transcriptomics to place IAld in the context of Wnt signaling pathway and PGD2 in the context of Ca2+ signaling pathway. They validated the effects of these two metabolites both in vitro and in vivo. It is an important work to expand the knowledge of ruminal development mechanism. The experiments and multi-omics techniques used by authors are comprehensive and provide strong evidence of IAld and PGD2 to be involved in ruminal development. The major issues include the lack of great details in method description, the lack of clarity in data presentation, and the loose link between microbial species and IAld/PGD2 production. Authors also should provide codes and data for better assessment.

Major comments:

1. Authors did a random forest analysis with very minimum description of their method beyond the package they used. Was binary or multi-class classification performed? What is the overall performance (e.g., accuracy) of the RF model(s)? Codes need to be provided.

2. The method description of the metagenomic analysis lacks great details: Which tool and reference database did authors use to calculate relative abundances? What percentage of reads were classified? How many genes were identified and used to normalize for TPM calculation? Codes should be provided.

3. Authors demonstrated that Bifidobacterium could convert tryptophan to IAld and Candida albicans could convert ARA to PGD2. However, the key genes including ArAT, PLA2, and PLB were only associated with certain feeding method but not associated with these particular species. Authors should demonstrate that those genes are present in the microbial genomes of these species, or present evidence that those genes are conserved in these species. At the very least, this should be discussed as a limitation of their analysis.

4. Fig. 3g demonstrated a significant correlation between PGD2 level and Candida albicans abundance. However, the three samples with the highest abundance of Candida albicans seem to be outliers. Authors should demonstrate the correlation was not purely driven by these samples.

5. Authors identified differentially expressed genes (DEGs) by comparing between MC, MH, and MHC groups. However, the functional analysis should be done for upregulated and downregulated genes separately and for different comparisons separately. Authors should also discuss other significant KEGG pathways, especially "Leukocyte transendothelial migration" and "Rap1 signaling pathway", which are more significant than Wnt signaling pathway.

6. Fig. 5b, e authors should provide source data and discuss what procedure was used to correct for multiple comparisons.

7. What type of correlation was conducted for correlation analysis throughout the manuscript (e.g., Pearson)? Please specify.

8. Authors need to provide codes and data for better assessment of their analysis and results.

Minor comments:

1. On lines 96-98, authors claimed that corn-soybean starter diet had the thickest rumen epithelia. However, cofeeding of corn-soybean starter and alfalfa hay did not result in an increased thickness of rumen epithelia (Fig. 1e). Authors should discuss this result.

2. Figure 1b lacks the legend to describe the error bars. Authors need to explain why the error bars only have the upper halves and show individual data points. The discrepancy of sample size in Fig1a-b (n = 10) and Fig1c-e (n = 8) should also be explained.

3. On lines 136, authors claimed that MC/MHC groups have the lowest tryptophan, but MH group is the lowest group based on Fig. 2g. Similarly, on line 141, MC and MHC groups have the highest linoleic acid instead of MH group as claimed by authors.

4. On lines 176-178, authors concluded "corn-soybean starter introduction contributed to the degradation of tryptophan into serotonin and indole pyruvate", which is an overstatement based on only gene abundances without measuring the end products. So is the concluding statement for the milk feeding.

5. Lines 800-802 describe two linear mixed models supposedly used in Fig. 1B. Authors should clarify which model was used to generate p-values in Fig. 1B and whether age was included as a fixed effect or random effect for the second model.

6. Authors mentioned the top 50 differential metabolites but less than 50 are shown in Fig. S1.

7. Some acronyms should be expanded to their full names when being mentioned for the first time. For example, PCA on line 119, KEGG on line 121.

8. All the significant pathways shown in Fig. 2b should be labeled.

9. Fig. 2c color bar needs a legend.

10. Fig. 2d color bar has a continuous scale, but the order of importance is discreet.

11. Since Fig. 3b shows relative abundances, the "enrichment" of bacteria in MC and MHC groups can also just be the reflection of the depletion of archaea in these groups. Authors need to discuss this clearly.

12. References for the following tools/databases are missing: CD-HIT, KEGG.

13. Gene names need to be italicized for ArAT on line 181, IAId on line 183.

14. Fig. 3d,e,g,h legends say relative abundance, which is usually represented by percentages, but the axis label says TPM, which is often used for genes instead of microbial abundances.

15. Fig. 3c, f error bars did not show lower limits. Why?

16. Fig. 3e y-axis has a breakpoint, but the upper half's lowest value is not shown and the breakpoint reference line on the bars is confusing. Authors could add reference lines at the break point or convert to log-scale.

17. On line 189, "relative abundance" is not appropriate to describe gene abundances represented by TPM values.

18. Grammar mistake on line 281: "were significantly enriched".

19. Typo on line 310, G2/GM phase should be G2/M.

20. An incomplete sentence on line 422.

21. Typo on line 503. Delete "are".

22. Fig. 7 legend describes n=8 in each group, but only six data points are plotted in boxplots and bar plots.

**Reviewer 2**

**Were you able to assess all statistics in the manuscript, including the appropriateness of statistical tests used?**

Yes, and I have assessed the statistics in my report.

**Comments to author:**

This article reported some information about nutrient-microbiome-host interactions in lambs. The authors found that IAld and PGD2 derived from early rumen microbiota were effective promoters of rumen development. The paper is of great interest and well written, however, I do have some comments on the paper.

1.How was the sample size considered for each group in the experiment, and what statistical method was used to determine the sample size in each group?

2.Line 136. The description of 'The ruminal tryptophan level was lowest in lambs with corn-soybean starter introduction' seems wrong according to Fig. 2g. Lambs with corn-soybean starter introduction (MC group) was blue in Fig .2g, however, the lowest was orange (MH group) in Fig .2g. The same problem was in Line 141-142 'Meanwhile, the lambs with alfalfa hay introduction had the highest level of ruminal linoleic acid and gamma-linolenic acid (Fig. 2g)'.

3.The color bar in Fig. 2d showed that the value from low to high was a kind of continuous value, while the colors in four groups were discrete. What were the values in the four groups in Fig. 2d? It had some risks to be believed that this picture was not manipulated.

4.There was no color bar in Fig. 2b.

5.Line 270. Why did not you mention 'Leukocyte transendothelial migration' and 'Rap1 signaling pathway', directly mentioned 'Wnt signaling pathway' in Fig. 5a? Why did you have such a choice?

6.'IAld and PGD2 derived from early rumen microbiota were effective promoters of rumen development' was a good story. However, were there any other possibilities, such as the digestive products of alfalfa or corn-soybean were effective promoters of rumen development? Please explain.

7.Line 328. 'This confirmed that the Ca2+/CAMK2 signaling pathway play a decisive role in the proliferation of ruminal smooth muscular cells', I think the conclusion is too strong. Is it the only determining factor? Could it work in other ruminants such as cows, goats and camels?

8.Please highlight the biggest discovery or significant advance in your research compared to previous studies in the Discussion section, and shorten the Discussion section because it was too long.

9.The welfare of experimental animals is an important aspect of animal experimental research in current scientific community. Please provide a supplementary description of your measures related to the animal welfare during your experiment.

10.All the raw data (Metabolites, Metagenome, Transcriptome) of this article should be uploaded into some public databases and be available for other researchers.

**Authors Response**

**Point-by-point responses to the reviewers’ comments:**

**Reviewer #1:**

**Comment 1:** Authors did a random forest analysis with very minimum description of their method beyond the package they used. Was binary or multi-class classification performed? What is the overall performance (e.g., accuracy) of the RF model(s)? Codes need to be provided.

***Response:*** *We thank the reviewer’s valuable comment. We are sorry that we miss the description of this detail in the original version. In the present study, a random forest classification was performed using the Random Forest package. The overall error rate is 0. We have provided the codes in the GitHub public database (https://github.com/sundm128/Genome-2023). We have also added the detailed description of random forest analysis as reading “In the present study, the random forest was used to select differentiating biomarkers among the different groups. A random forest classification was* *performed using the Random Forest package in R (version 3.6.2) with 500 trees and 10-fold* *cross-validation to obtain robust estimates of the generalization error and feature importance. We used out-of-bag (OOB) error rate to measure the performance of the model. Our dataset was partitioned into a training set (including 70% of the samples) and a validation set (including the remaining 30%). We have provided the codes in the GitHub public database (*[*https://github.com/sundm128/Genome-2023*](https://github.com/sundm128/Genome-2023)*). Prism 8 (GraphPad Software, La Jolla, CA, USA) was used to construct a graph of TOP 20 metabolites based on Mean decrease accuracy value”* ***(Page 52, Line 726-736)****. We also added the description of accuracy as reading “Moreover, we performed a random forest classifier using the level of metabolites as variables (100% classification accuracy)”* ***(Page 10, Line 141-142)*** *in the revised manuscript.*

**Comment 2:** The method description of the metagenomic analysis lacks great details: Which tool and reference database did authors use to calculate relative abundances? What percentage of reads were classified? How many genes were identified and used to normalize for TPM calculation? Codes should be provided.

***Response:*** *We really appreciate for reviewer’s warm work earnestly and agree with these comments. According to the reviewer’s suggestion, we have added the detailed method* *description of the metagenomic analysis as reading “Entries in all the gene catalogs were subjected to taxonomic and functional assignment using DIAMOND (v.0.9.22; Buchfink B, 2015) based on BLASTP searches against the NCBI-NR (October 2018; approximately 550 M sequences) and KEGG (v.90.0; Kanehisa M, 2004) databases (parameter: --evalue 0.00001 --max-target-seqs 10). The high-quality reads from each sample were aligned against the gene catalogs using BWA-MEM (v.0.7.17; Li H, 2010), and abundance profiles of genes (alignment length ≥ 50 bp and sequence identity > 95%) were calculated in transcripts per million (TPM; Wagner GP, 2012), with corrections for variations in gene length and mapped reads per sample. TPM is calculated as:*

$$TPM=\frac{N\text{g}}{L\text{g}}\times\frac{1}{\text{Σj}\frac{N\text{j}}{L\text{j}}}\times10$$

*where Ng is the read count, i.e., the average number of reads mapped to the g gene; and Lg is the gene length, i.e., the number of nucleotides in the g gene. The index j stands for the set of all genes determined in a catalog, and g is an index indicating a particular gene (Wagner GP, 2012). The relative abundances of taxa and KOs were calculated from the abundances of annotated genes (Li J, 2014). Briefly, for the taxonomic profiles, we used phylogenetic assignment of each annotated gene from the rumen microbial gene catalog and summed the relative abundances of genes from the same phylum or genus to produce the abundance of each phylum or genus. The profile of each KO was calculated using the same process. The relative abundance of a KEGG pathway was calculated from the summation of the relative abundances of its contained KOs. The parameters or codes of software and packages mentioned were descripted in the previous studies (Lin L, 2019; Xie F, 2021)” in the revised manuscript* ***(Page 54, Lin753-774).***

*Moreover, we added more general data about the metagenomic analysis as reading “We generated a nonredundant rumen microbial gene catalog that comprises 12,707,419 genes. According to currently available databases, 48.93% of rumen microbial gene catalog were taxonomically classified as originating from bacteria (47.21%), archaea (1.02%), eukaryote (0.58%) and virus (0.04%), and 41.87% (5,320,684) of rumen microbial gene catalog were annotated to KEGG orthologous groups (KOs)” in the results section of revised manuscript* ***(Page 14, Lin179-184)****.*

***Reference***:

*Buchfink B, Xie C, Huson DH: Fast and sensitive protein alignment using DIAMOND. Nat Methods 2015, 12:59-60.*

*Kanehisa M, Goto S, Kawashima S, Okuno Y, Hattori M: The KEGG resource for deciphering the genome. Nucleic Acids Res 2004, 32:D277-280.*

*Li H, Durbin R: Fast and accurate long-read alignment with Burrows-Wheeler transform. Bioinformatics 2010, 26:589-595.*

*Wagner GP, Kin K, Lynch VJ: Measurement of mRNA abundance using RNA-seq data: RPKM measure is inconsistent among samples. Theory in Biosciences 2012, 131:281-285.*

*Li J, Jia H, Cai X, Zhong H, Feng Q, Sunagawa S, et al: An integrated catalog of reference genes in the human gut microbiome. Nat Biotechnol 2014, 32:834-841.*

*Lin L, Xie F, Sun D, Liu J, Zhu W, Mao S: Ruminal microbiome-host crosstalk stimulates the development of the ruminal epithelium in a lamb model. Microbiome 2019, 7:83.*

*Xie F, Jin W, Si H, Yuan Y, Tao Y, Liu J, et al: An integrated gene catalog and over 10,000 metagenome-assembled genomes from the gastrointestinal microbiome of ruminants. Microbiome 2021, 9:137.*

**Comment 3:** Authors demonstrated that Bifidobacterium could convert tryptophan to IAld and *Candida albicans* could convert ARA to PGD2. However, the key genes including ArAT, PLA2, and PLB were only associated with certain feeding method but not associated with these particular species. Authors should demonstrate that those genes are present in the microbial genomes of these species, or present evidence that those genes are conserved in these species. At the very least, this should be discussed as a limitation of their analysis.

***Response:*** *Thanks for reviewer’s valuable comments and agree with this important suggestion. In the present study, we have validated the critical roles of IAld and PGD2 in the rumen development in vitro and in vivo. We also further confirmed that Bifidobacterium pseudolongum and Candida albicans have the ability to produce IAld and PGD2, respectively (Figure 4). However, to date, the research information on the metabolic process and key genes involved in IAld production is very limited. ArAT gene is one of known genes involved in IAld production (Zelante T, 2013), but we did not identify the ArAT gene in the Bifidobacterium pseudolongum genome, implying that Bifidobacterium pseudolongum may produce IAld through other unknown co-metabolizing pathways, which is need to be further explored. Moreover, a previous study reported that PLA2 and PLB, which are released by fungi, play important roles in the conversion of phospholipids into arachidonic acid (Kohler GA, 2006). However, detailed biosynthetic pathways of PGD2 from arachidonic acid are still unavailable in fungi. We have also discussed this limitation as reading “our study demonstrated that Bifidobacterium pseudolongum is able to convert tryptophan into IAld and Candida albicans is a key producer for PGD2, but the key conversion pathways and microbial enzyme genes involved in this process are need to be further clarified” in the revised manuscript* ***(Page 36, Line 491-494)****. Thanks again for your insightful comment and we will take this important suggestion into consideration in our future research.*

***Reference****:*

*Zelante T, Iannitti RG, Cunha C, De Luca A, Giovannini G, Pieraccini G, et al: Tryptophan Catabolites from Microbiota Engage Aryl Hydrocarbon Receptor and Balance Mucosal Reactivity via Interleukin-22. Immunity 2013, 39:372-385.*

*Kohler GA, Brenot A, Haas-Stapleton E, Agabian N, Deva R, Nigam S: Phospholipase A2 and phospholipase B activities in fungi. Biochim Biophys Acta 2006, 1761:1391-1399.*

**Comment 4:** Fig. 3g demonstrated a significant correlation between PGD2 level and *Candida albicans* abundance. However, the three samples with the highest abundance of *Candida albicans* seem to be outliers. Authors should demonstrate the correlation was not purely driven by these samples.

***Response:*** *Thanks for reviewer’s valuable comments. We recalculated the correlation between PGD2 level and Candida albicans abundance excluding the three samples with the highest abundance of Candida albicans, and the positive correlation still exists (P = 0.007, r = 0.5939). Thus, this positive correlation result was not purely driven by the three samples with the highest abundance of Candida albicans.*

**Comment 5:** Authors identified differentially expressed genes (DEGs) by comparing between MC, MH, and MHC groups. However, the functional analysis should be done for upregulated and downregulated genes separately and for different comparisons separately. Authors should also discuss other significant KEGG pathways, especially "Leukocyte transendothelial migration" and "Rap1 signaling pathway", which are more significant than Wnt signaling pathway.

***Response:*** *Thanks for reviewer’s valuable comments. We have added the KEGG analysis of DEGs for different comparisons separately in the revised manuscript. Moreover, together with the comment of reviewer 2, we have added the discussion as reading “The DEGs associated with epithelial development biological process were significantly enriched in the leukocyte transendothelial migration, rap1 signaling pathway, and Wnt signaling pathway (Fig. 5a, Additional file 3: Table S2). The Leukocyte transendothelial migration was widely recognized as the key elements of both innate and adaptive immunity of the vessel wall and the underlying tissue (Muller, 2011). By now it was recognized that Rap1 had the main function in induction of cell adhesion, either to other cells or to extracellular matrix (Boettner and Van, 2009). Wnt signaling pathway has been demonstrated to play important roles in gut epithelial development and maintaining the proliferative compartment of the adult gut epithelium in multicellular animals (Steinhart and Angers, 2018). Inspiringly, we investigated the role of Wnt pathway in rumen epithelial development and analyzed the correlation between ruminal epithelial thickness and gene expression related to the Wnt signaling pathway” in the revised manuscript* ***(Page 22, Line 287-298)****.*

***Reference***:

*Muller WA: Mechanisms of leukocyte transendothelial migration. Annu Rev Pathol 2011, 6:323-344.*

*Boettner B, Van Aelst L: Control of cell adhesion dynamics by Rap1 signaling. Curr Opin Cell Biol 2009, 21:684-693.*

*Steinhart Z, Angers S: Wnt signaling in development and tissue homeostasis. Development 2018, 145.*

**Comment 6:** Fig. 5b, e authors should provide source data and discuss what procedure was used to correct for multiple comparisons.

***Response:*** *Thanks for reviewer’s valuable comments. We have added the source data in the* ***Additional file 4: Table S3 and Additional file 6: S5*** *and also recorrected the p value by FDR for multiple comparisons* ***(Fig. 5b, e)****.*

**Comment 7**. What type of correlation was conducted for correlation analysis throughout the manuscript (e.g., Pearson)? Please specify.

***Response:*** *Thanks for reviewer’s valuable comments. In the present study, Spearman’s correlation was used for correlation analysis throughout the manuscript. We have added the corresponding description in the figure legends.*

**Comment 8**. Authors need to provide codes and data for better assessment of their analysis and results.

***Response:*** *We really appreciate for reviewer’s valuable comment. All the raw data (Metabolome, Metagenome, Transcriptome) of this article have uploaded into public databases. We have added the corresponding description as reading “The metagenomic datasets analyzed in this study have been deposited in the CNGB Sequence Archive (CNSA) of the China National GeneBank DataBase (CNGBdb) under accession number CNP0005016 (https://db.cngb.org/search/project/CNP0005016/). The metabolomic datasets analyzed in this study have been deposited in the CNSA of the CNGBdb under accession number CNP0005042 (https://db.cngb.org/search/project/CNP0005042/) and CNP0004958 (https://db.cngb.org/search/project/CNP0004958/). The transcriptomic datasets analyzed in this study are available at National Center for Biotechnology Information under accession numbers PRJNA881929 (*[*https://www.ncbi.nlm.nih.gov/bioproject/PRJNA881929)*](https://www.ncbi.nlm.nih.gov/bioproject/PRJNA881929))*” in the revision* ***(Page 72, Line 1054-1064)****. Moreover, we have provided the codes in the GitHub public database (https://github.com/sundm128/Genome-2023) and added the references of codes for metagenomics amalysis in the revised manuscript.*

**Minor comments:**
**Comment 1**. On lines 96-98, authors claimed that corn-soybean starter diet had the thickest rumen epithelia. However, cofeeding of corn-soybean starter and alfalfa hay did not result in an increased thickness of rumen epithelia (Fig. 1e). Authors should discuss this result.

***Response:*** *Thanks for reviewer’s valuable comments. We have added corresponding discussion as reading “Compared with corn-soybean starter introduction, cofeeding of corn-soybean starter and alfalfa hay decreased the thickness of rumen epithelia (Figure 1e), while increased the length of ruminal epithelial papillae (Figure 1d). Previous study indicated the elongation of gastrointestinal epithelium through regulation cell division, cell elongation, and cell rearrangement (Andrew DJ, 2010). Thus, we speculate that cofeeding of corn-soybean starter and alfalfa hay facilitated the ruminal epithelial cell division orientation for the growth of ruminal epithelial papillae.” in the revision* ***(Page 7, Line 98-105)****.*

***Reference:***

*Andrew DJ, Ewald AJ: Morphogenesis of epithelial tubes: Insights into tube formation, elongation, and elaboration. Developmental Biology 2010, 341:34-55.*

**Comment 2**. (1) Figure 1b lacks the legend to describe the error bars. Authors need to explain why the error bars only have the upper halves and show individual data points. The discrepancy of sample size in Fig1a-b (n = 10) and Fig1c-e (n = 8) should also be explained.
***Response:*** *We are sorry for this confusion. The Figure 1b was produced by the Graphpad PRISM software. In order to prevent the lower halves cover the data line, we set up to display only the upper halves. The Graphpad website explains why Prism does not plot some error bars. Please see the figure as below. According to the reviewer’s suggestion, we have added both the upper and lower halves in the Fig 1b of revised manuscript.*

(2) The discrepancy of sample size in Fig1a-b (n = 10) and Fig1c-e (n = 8) should also be explained.

***Response:*** *Thanks for reviewer’s valuable comments. Forty healthy Hu lambs (11-day-old) were used in the animal feeding experiment. However, at 42 d of age, eight healthy lambs per group were randomly select for slaughter. We have added this detail as reading “At 42 d of age, eight healthy lambs per group were randomly select to be stunned using electric shock and sacrificed by exsanguination” in the revised manuscript* ***(Page 40, Line 542-544)****.*

**Comment 3**. On lines 136, authors claimed that MC/MHC groups have the lowest tryptophan, but MH group is the lowest group based on Fig. 2g. Similarly, on line 141, MC and MHC groups have the highest linoleic acid instead of MH group as claimed by authors

***Response:*** *Thanks for reviewer’s valuable comments. We have revised it as reading “The level of IAld precursor tryptophan is highest in lambs with only milk intake (Fig. 2g).”* ***(Page 11, Line 145-146)****; “Meanwhile, lambs with corn-soybean starter introduction exhibited the highest level of ruminal linoleic acid and gamma-linolenic acid”* ***(Page 11, Line 149-150)*** *in the revised manuscript.*

**Comment 4**. On lines 176-178, authors concluded "corn-soybean starter introduction contributed to the degradation of tryptophan into serotonin and indole pyruvate", which is an overstatement based on only gene abundances without measuring the end products. So is the concluding statement for the milk feeding.

***Response:*** *We are really appreciated reviewer’s valuable comments. According to the reviewer’s suggestions, we have removed these overstatements throughout the manuscript.*

**Comment 5**. Lines 800-802 describe two linear mixed models supposedly used in Fig. 1B. Authors should clarify which model was used to generate p-values in Fig. 1B and whether age was included as a fixed effect or random effect for the second model

***Response:*** *We thank the reviewer’s valuable comment. We have added the corresponding descriptions as reading “For body weight and daily nutrient intake of lambs, the statistical analysis was performed using the mixed linear model. The treatment, age, and their interaction were treated as fixed factors and the lamb was considered as a random effect. When significant differences observed among different treatments, a multiple comparison of body weight at 42 d was conducted based on one-way ANOVA analysis” in the revised manuscript* ***(Page 58, Line 825-829)****.*

**Comment 6**. Authors mentioned the top 50 differential metabolites but less than 50 are shown in Fig. S1.

***Response:*** *We are sorry for this confusion. In the original version, we just presented the top metabolites belonging to carbohydrate metabolites, amino acid metabolites, lipid metabolites, and plant active substance metabolites, but the other 10 metabolites not belonging to these four main functional categories did not present. According to the reviewer’s comment, we have included all the top 50 metabolites into new Fig.1S in the revised manuscript.*

**Comment 7.** Some acronyms should be expanded to their full names when being mentioned for the first time. For example, PCA on line 119, KEGG on line 121.

***Response:*** *Thanks for reviewer’s valuable comments. We have checked all the acronyms and added their full names when being mentioned for the first time in the revised manuscript.*

**Comment 8.** All the significant pathways shown in Fig. 2b should be labeled.

***Response:*** *Thanks for reviewer’s valuable comments. We have labeled all the significant pathways in the revised manuscript* ***(Figure 2b).***

**Comment 9.** Fig. 2c color bar needs a legend.

***Response:*** *Thanks for reviewer’s valuable comments. We have added the legend of color bar (r value) in the revised manuscript* ***(Fig. 2c)****.*

**Comment 10.** Fig. 2d color bar has a continuous scale, but the order of importance is discreet.

***Response:*** *We are sorry for this confusion. The colored boxes on the right indicate the relative level of the corresponding metabolite in each group. We have added the description and revised the color bar in the Figure 2d legend.*

**Comment 11.** Since Fig. 3b shows relative abundances, the "enrichment" of bacteria in MC and MHC groups can also just be the reflection of the depletion of archaea in these groups. Authors need to discuss this clearly.

***Response:*** *Thanks for reviewer’s valuable comments. We have added corresponding discussion as reading “We observed that corn-soybean-fed lambs were frequently enriched with ruminal bacteria, which may just be the reflection of the depletion of ruminal archaea, while alfalfa hay-fed lambs were more occupied with eukaryota in the rumen” in the revised manuscript* ***(Page 14, Line 186-189)****.*

**Comment 12.** References for the following tools/databases are missing: CD-HIT, KEGG.

***Response:*** *Thanks for reviewer’s valuable comments. We have added the references in the revised manuscript* ***(Page 54, Line 753-774)****.*

**Comment 13.** Gene names need to be italicized for ArAT on line 181, IAId on line 183.

***Response:*** *Thanks for reviewer’s comments. We have changed it in the revised manuscript* ***(Page 15, Line 193)****.*

**Comment 14.** Fig. 3d,e,g,h legends say relative abundance, which is usually represented by percentages, but the axis label says TPM, which is often used for genes instead of microbial abundances.

***Response:*** *Thanks for reviewer’s valuable comments. We have recalculated and replaced the TPM with percentage (%) in the revised manuscript* ***(Fig. 3d, e, g, h)****.*

**Comment 15.** Fig. 3c, f error bars did not show lower limits. Why?

***Response:*** *We are sorry for this confusion. The Figure 3c, f were produced by the PRISM software. We only displayed the upper halves (left figure as below) in the original draft. According to the reviewer’s comment, we have added both upper halves and lower halves* ***(Fig. 3c, f).***

**Comment 16.** Fig. 3e y-axis has a breakpoint, but the upper half's lowest value is not shown and the breakpoint reference line on the bars is confusing. Authors could add reference lines at the break point or convert to log-scale.

***Response:*** *Thanks for reviewer’s valuable comments. We have added the upper half’s lowest value of Fig. 3e in the revised manuscript.*

**Comment 17.** On line 189, "relative abundance" is not appropriate to describe gene abundances represented by TPM values.

***Response:*** *Thanks for reviewer’s valuable comments. We have rephrased it in the revised manuscript.*

**Comment 18.** Grammar mistake on line 281: "were significantly enriched".

***Response:*** *Thanks for reviewer’s valuable comments. We have revised it in the revised manuscript* ***(Page 23, Line 313)****.*

**Comment 19.** Typo on line 310, G2/GM phase should be G2/M.

***Response:*** *Thanks for reviewer’s comments. We have revised it in the revised manuscript* ***(Page 26, Line 346)****.*

**Comment 20.** An incomplete sentence on line 422.

***Response:*** *Thanks for reviewer’s comments. Together with our response to comment 4 and reviewer 2, we have removed this sentence in the revision.*

**Comment 21.** Typo on line 503. Delete "are".

***Response:*** *Thanks for reviewer’s valuable comments. We have deleted it in the revised manuscript* ***(Page 37, Line 494)****.*

**Comment 22.** Fig. 7 legend describes n=8 in each group, but only six data points are plotted in boxplots and bar plots.

***Response:*** *Thanks for reviewer’s valuable comments. We are sorry for this confusion. In the present study, 24 lambs with good adaptation to artificial nursing were randomly assigned to three groups with the following treatments: normal saline infusion (Con, n = 8), IAld infusion (IAld, n = 8), and PGD2 infusion (PGD2, n = 8). During the feeding and oral infusion period, lambs with diarrhea, pneumonia and aphtha were excluded. At 42 d of age, six healthy lambs per group were slaughtered and the ruminal epithelium and muscle tissue were collected for morphology analysis and RNA extraction. We have added the details in the section of methods in the revised manuscript* ***(Page 43, Line 579-583)****.*

**Reviewer 2**

**Comment 1**. How was the sample size considered for each group in the experiment, and what statistical method was used to determine the sample size in each group?

***Response:*** *Thanks for reviewer’s valuable comments. Before the experiment, based on one-way ANOVA, we used G*Power software (version 3.1.9.6) for the power analysis with following variables; the power = 0.8, significant level = 0.05, and effect size = 0.75 in the experiment. The effect size was estimated based on the results from preliminary study. Hence, the required minimal sample size was six lambs per group. After the experiment, using G*Power, we perform a post-hoc analysis to determine the effect size for animal experiment and our samples could detect with adequate power (80%) based on an alpha = 0.05 (uncorrected p), based on F-test using one-way ANOVA. We have added the description as reading “Using G*Power (3.1.9.6), we performed a post-hoc analysis to determine the effect size for animal experiment and our samples could detect with adequate power (80%) based on an alpha = 0.05, and F-test using one-way ANOVA” in the revised manuscript* ***(Page 59, Line 840-844)****.*

**Comment 2**. Line 136. The description of 'The ruminal tryptophan level was lowest in lambs with corn-soybean starter introduction' seems wrong according to Fig. 2g. Lambs with corn-soybean starter introduction (MC group) was blue in Fig .2g, however, the lowest was orange (MH group) in Fig .2g. The same problem was in Line 141-142 'Meanwhile, the lambs with alfalfa hay introduction had the highest level of ruminal linoleic acid and gamma-linolenic acid (Fig. 2g)'.

***Response:*** *We really appreciate for reviewer’s comments. Indeed, it was a mistake. we have carefully checked it and revised it as reading “The level of IAld precursor tryptophan is highest in lambs with only milk intake (Fig. 2g).”* ***(Page 11, Line 146-146)****; “Meanwhile, lambs with corn-soybean starter introduction exhibited the highest level of ruminal linoleic acid and gamma-linolenic acid”* ***(Page 11, Line 149-150)*** *in the revised manuscript.*

**Comment 3**. The color bar in Fig. 2d showed that the value from low to high was a kind of continuous value, while the colors in four groups were discrete. What were the values in the four groups in Fig. 2d? It had some risks to be believed that this picture was not manipulated.

*Response: We thank the reviewer’s valuable comment and sorry for this confusion. In the present study, Prism 8 (GraphPad Software, La Jolla, CA, USA) was used to construct a* *graph of TOP 20 metabolites based on Mean decrease accuracy value. We used four kinds of color to distinguish the different level of metabolites among four groups. To clarify, we have added the description as reading “The colored boxes on the right represented the level of the corresponding metabolites in each group”. We have also revised the color bar in the Figure 2d legend.*

**Comment 4**. There was no color bar in Fig. 2b.

*Response: We are sorry for this confusion. We have added the color bar in the Fig.2b in the revised manuscript.*

**Comment 5**. Line 270. Why did not you mention 'Leukocyte transendothelial migration' and 'Rap1 signaling pathway', directly mentioned 'Wnt signaling pathway' in Fig. 5a? Why did you have such a choice?

***Response:*** *Thanks for reviewer’s valuable comments. Wnt pathway has been demonstrated to play important roles in gut epithelial development and maintaining the proliferative compartment of the adult gut epithelium in multicellular animals. Inspiringly, we selected Wnt pathway. Together with the comment of reviewer 1, we have added the discussion as reading “The DEGs associated with epithelial development biological process were significantly enriched in the leukocyte transendothelial migration, rap1 signaling pathway, and Wnt signaling pathway* ***(Fig. 5a, Additional file 3: Table S2)****. The Leukocyte transendothelial migration was widely recognized as the key elements of both innate and adaptive immunity of the vessel wall and the underlying tissue (Muller, 2011). By now it was recognized that Rap1 had the main function in induction of cell adhesion, either to other cells or to extracellular matrix (Boettner and Van, 2009). Wnt signaling pathway has been demonstrated to play important roles in gut epithelial development and maintaining the proliferative compartment of the adult gut epithelium in multicellular animals (Steinhart and Angers, 2018). Inspiringly, we investigated the role of Wnt pathway in rumen epithelial development and analyzed the correlation between ruminal epithelial thickness and gene expression related to the Wnt signaling pathway” in the revised manuscript* ***(Page 22, Line 287-298)****.*

***Reference***:

*Muller WA: Mechanisms of leukocyte transendothelial migration. Annu Rev Pathol 2011, 6:323-344.*

*Boettner B, Van Aelst L: Control of cell adhesion dynamics by Rap1 signaling. Curr Opin Cell Biol 2009, 21:684-693.*

*Steinhart Z, Angers S: Wnt signaling in development and tissue homeostasis. Development 2018, 145.*

**Comment 6**. 'IAld and PGD2 derived from early rumen microbiota were effective promoters of rumen development' was a good story. However, were there any other possibilities, such as the digestive products of alfalfa or corn-soybean were effective promoters of rumen development? Please explain.

***Response:*** *We really appreciate for this important suggestion by the reviewer and agree with this comment. Rumen mainly relies on colonization microorganisms to ferment the plant feed (Mizrahi I, 2021). Degradation and metabolism of plant feed is accomplished through synchronous activities of the multitude of microbial enzymes present in the rumen (Wang Y, 2002). Thus, ruminal microbial metabolites may play crucial roles in rumen development (Malmuthuge N, 2019). Many previous studies have demonstrated that ruminal microbiota-derived VFA can promote the development of rumen epithelium (Beiranvand H, 2014; Górka P, 2011). In the present study, we cannot fully exclude the detected ruminal IAld derived from diet or their digestive products, but we did demonstrate that the enriched Bifidobacterium pseudolongum in lambs fed corn-soybean starter has the ability to produce IAld. Moreover, based on the Random Forest analysis, except for the IAld and PGD2, the other ruminal characteristic metabolites in lambs fed corn-soybean starter (such as oleic acid, daidzein and 5-hydroxyindoleacetic) or in lambs fed alfalfa hay (nandrolone, enterolactone, and salicylic acid) may also contribute to rumen development, which need to be further explored. We have added the corresponding discussion in the revision* ***(Page 37, Line 502-504)****. Thank you again for your insightful suggestion and we will also take this important comment into consideration in our future research.*

***Reference：***

*Mizrahi I, Wallace R J, Moraïs S. The rumen microbiome: balancing food security and environmental impacts[J]. Nature Reviews Microbiology, 2021, 19(9): 553-566.*

*Wang Y, McAllister T A. Rumen microbes, enzymes and feed digestion-a review[J]. Asian-Australasian Journal of Animal Sciences, 2002, 15(11): 1659-1676.*

*Malmuthuge N, Liang G, Guan L L. Regulation of rumen development in neonatal ruminants through microbial metagenomes and host transcriptomes[J]. Genome Biology, 2019, 20(1): 1-16.*

*Beiranvand H, Ghorbani G R, Khorvash M, et al. Interactions of alfalfa hay and sodium propionate on dairy calf performance and rumen development[J]. Journal of Dairy Science, 2014, 97(4): 2270-2280.*

*Górka P, Kowalski Z M, Pietrzak P, et al. Effect of method of delivery of sodium butyrate on rumen development in newborn calves[J]. Journal of Dairy Science, 2011, 94(11): 5578-5588.*

**Comment 7.** Line 328. 'This confirmed that the Ca2+/CAMK2 signaling pathway play a decisive role in the proliferation of ruminal smooth muscular cells', I think the conclusion is too strong. Is it the only determining factor? Could it work in other ruminants such as cows, goats and camels?

***Response:*** *Thanks for reviewer’s valuable comments. We have revised it as reading “This confirmed that the Ca^2+^/CAMK2 signaling pathway play a crucial role in the proliferation of ruminal smooth muscular cells.” in the revised manuscript* ***(Page 27, Line 364-366)****.*

**Comment 8.** Please highlight the biggest discovery or significant advance in your research compared to previous studies in the Discussion section, and shorten the Discussion section because it was too long.

***Response:*** *Thanks for reviewer’s valuable comments. We have extensive revised and shortened the discussion section in the revised manuscript.*

**Comment 9.** The welfare of experimental animals is an important aspect of animal experimental research in current scientific community. Please provide a supplementary description of your measures related to the animal welfare during your experiment.

***Response:*** *Thanks for reviewer’s valuable comments. We have provided a supplementary description of the measures related to the animal welfare during experiment* ***(Additional file 7)*** *and we have also added the corresponding description as reading “The detailed description of animal welfare during lamb experiment was presented in Additional file 7” in the revision* ***(Page 40, Line 529; Page 41, Line 556)****.*

**Comment 10.** All the raw data (Metabolites, Metagenome, Transcriptome) of this article should be uploaded into some public databases and be available for other researchers.

***Response:*** *Thanks for reviewer’s valuable comments. All the raw data (Metabolites, Metagenome, Transcriptome) of this article have uploaded into public databases. We have added more details as reading “The metagenomic datasets analyzed in this study have been deposited in the CNGB Sequence Archive (CNSA) of the China National GeneBank DataBase (CNGBdb) under accession number CNP0005016 (https://db.cngb.org/search/project/CNP0005016/). The metabolomic datasets analyzed in this study have been deposited in the CNSA of the CNGBdb under accession number CNP0005042 (https://db.cngb.org/search/project/CNP0005042/) and CNP0004958 (https://db.cngb.org/search/project/CNP0004958/). The transcriptomic datasets analyzed in this study are available at National Center for Biotechnology Information under accession numbers PRJNA881929 (https://www.ncbi.nlm.nih.gov/bioproject/PRJNA881929)” in the revision* ***(Page 72, Line 1054-1063)****.*

**Second round of review**

**Reviewer 1**

Authors have addressed most of my previous comments with a couple in need of further explanation. Authors also need to make sure all the additional data included in the revised manuscript are properly labeled.

1. Authors didn’t fully address my previous Major Comment #5 regarding the functional KEGG pathways enriched in upregulated and downregulated genes, separately. Moreover, even though authors performed additional analyses and showed the significant pathways for each pairwise comparison between MC, MH, and MHC, the results in new Figs. S3 and S4 are hard to make sense, especially when metabolic pathways are one of the top enriched pathways in each comparison but disappear when combining all DEGs. Authors need to explain this and discuss the different pathways enriched in individual comparisons and the combined comparison.

2. Authors addressed my previous Minor Comment #8 by adding annotations to more circles in Fig. 2b, which I suppose are significant pathways. However, looking at the -log10(P) on the y-axis, none of these pathways seems passing FDR < 0.05, which requires -log10(P) > 1.3. In addition, the dot size lacks legend.

3. Authors should check the places where they used “Figs” to refer to only a single figure throughout the manuscript.

4. In Additional file 6, authors included many unreferenced tables.

5. Authors should make sure the gene names in Fig. 5b,e match the gene names included in Additional files 4 and 6.

6. Supplemental tables in Additional files 4 and 5 are mislabeled.

**Reviewer 2**

The revisions improve the manuscript. However, there remain additional limitations to the study design and analysis that should explicitly be mentioned in the manuscript for the benefit of the readership.

Minor comments:

1. Line 145, the ‘is’ should be ‘was’. Line 151, the ‘are’ should be ‘were’. Other parts of the manuscript also need to be checked.

2.Figures should be provided with vector images to avoid affecting clarity, as the current TIFF format lacks clarity.

3.Based on your findings, what potential impacts will it have on future animal husbandry or animal feed? Please explain.

**Authors Response**

**Point-by-point responses to the reviewers’ comments:**

**Response to reviewers**

**Reviewer #1:**

**Comment 1:** Authors didn’t fully address my previous Major Comment #5 regarding the functional KEGG pathways enriched in upregulated and downregulated genes, separately. Moreover, even though authors performed additional analyses and showed the significant pathways for each pairwise comparison between MC, MH, and MHC, the results in new Figs. S3 and S4 are hard to make sense, especially when metabolic pathways are one of the top enriched pathways in each comparison but disappear when combining all DEGs. Authors need to explain this and discuss the different pathways enriched in individual comparisons and the combined comparison.

***Response:*** *We thank the reviewer’s valuable comment. We are sorry that we did not fully address the reviewer’s concerns about Major Comment #5. According to reviewer’s suggestion, we have re-analyzed the functional KEGG pathways enriched in upregulated and downregulated genes for each pairwise comparison between MC, MH, and MHC* ***(Additional* *file 1: Fig. S4 and S6)*** *and have added these results as reading “Based on the functional KEGG pathways enrichment in upregulated and downregulated genes for each pairwise comparison between MC, MH, and MHC, we found that starchy corn-soybean starter introduction mainly enriched the rumen epithelial metabolism pathways, biosynthesis of amino acid, and glycerolipid metabolism (****Additional file 1: Fig. S4****), suggesting that rumen epithelial development was accompanied by enhancement of metabolic function. For the ruminal muscle transcriptome, a total of 140, 131, and 80 DEGs were obtained in MC versus MH, MHC versus MC, and MHC versus MH, respectively (Additional file 1: Fig. S2). Based on the KEGG analysis of DEGs in each comparison, we found that calcium signaling pathway and axon guidance were mainly enriched in these DEGs (Additional file 1: Fig. S5). In addition, we also analyzed the functional KEGG pathways enriched in upregulated and downregulated genes for each pairwise comparison between MC, MH, and MHC and found that alfalfa hay introduction mainly enhanced calcium signaling pathway, adipocytokine signaling pathway, and PPAR signaling pathway (Additional file 1: Fig. S6)”* ***(Page 21-22, Line 286-300)*** *in the revised manuscript.*

*Based on all the KEGG analysis for each pairwise comparison between MC, MH, and MHC (****Additional file 1: Fig. S4-S6****), it is hard to identify the specific signal pathways associated with rumen epithelial and muscular development. Thus, we need a clearer sense of the relationship between rumen development and signal pathway activation. Here, we mainly discerned the pathways associated with cell proliferation and development in the rumen epithelial or muscle samples. Thus, all DEGs among three groups in the rumen epithelial* or *muscle samples were used to conduct Gene Ontology (GO) biological processes enrichment analysis (****Additional file 3: Table S2 and Additional file 6: Table S5****). Then, we selected the specific DEGs involved in GO terms associated with epithelial or muscle development (****Additional file 4: Table S3 and Additional file 7: Table S6****) for further KEGG analysis. In the revised manuscript, we have revised it as reading “Based on all the KEGG analysis for each pairwise comparison between MC, MH, and MHC (Additional file 1: Fig. S3-S6), it was unable to discriminate the specific signal pathways associated with rumen epithelial and muscular development. Here, Gene Ontology (GO) biological processes enrichment analysis was conducted using 1017 DEGs obtained among three groups in the rumen epithelia (Additional file 3: Table S2). We found that epithelium development (GO:0060429),* *regulation of epithelial cell proliferation (GO:0050678), epithelial cell differentiation (GO:0030855), epithelial cell development (GO:0002064), negative regulation of epithelial cell proliferation* *(GO:0050680), epithelial cell proliferation (GO:0050673) were the significantly enriched biological process related to rumen epithelial development (Additional file 4: Table S3). Furthermore, these specific 70 DEGs belonging to biological process of epithelial development and cell proliferation (Additional file 4: Table S3) were selected and clustered for further KEGG analysis. It exhibited that these 70 DEGs were significantly enriched in the leukocyte transendothelial migration, Rap1 signaling pathway, and Wnt signaling pathway (Fig. 5a)”* ***(Page 22-23, Line 301-315)*** *and “To further discern the key pathways associated with rumen muscle development, a total of 291 DEGs in the rumen muscle obtained among three groups (Additional file 1: Fig. S2) were used to conduct GO biological processes enrichment analysis (Additional file 6: Table S5). We found that muscle contraction (GO:0006936), connective tissue development (GO:0061448), muscle system process (GO:0003012), smooth muscle contraction (GO:0006939), and muscle structure development (GO:0061061) were the significantly enriched biological process related to rumen muscular development (Additional file 7: Table S6). These specific 26 DEGs belonging to biological process of rumen muscular development (Additional file 7: Table S6) were selected and clustered for further KEGG analysis and it showed that the Ca^2+^ signaling pathway was the top significantly enriched pathway (Fig. 5d)”* ***(Page 24-25, Line 330-340)*** *in the revised manuscript.*

*We have also explained the limitations in the discussion section as reading “we have gained some important new findings, but due to the limitations of agriculture animal feeding and analysis technology, some issues still remain to be further explored.” in the revision (****Page 37, Line 501-503****). Thanks again for the reviewer’s important comment, we will take it consideration in our future research and make great efforts to overcome these limitations of analysis.*

**Comment 2:** Authors addressed my previous Minor Comment #8 by adding annotations to more circles in Fig. 2b, which I suppose are significant pathways. However, looking at the -log10(P) on the y-axis, none of these pathways seems passing FDR < 0.05, which requires -log10(P) > 1.3. In addition, the dot size lacks legend.

***Response:*** *We really appreciate for reviewer’s warm work earnestly and agree with these comments. Our previous enrichment analysis was conducted by Metaboanalyst 4.0, and the results showed that all pathways were not significant enrichment. With the update of analysis tools (below figure), we re-analyzed the data using Metaboanalyst 6.0 (https://www.metaboanalyst.ca/MetaboAnalyst/home.xhtml) and found that the P values of Linoleic acid metabolism and Tryptophan metabolism were 0.045 and 0.051, respectively. We have revised the Fig 2b and added the dot size legend in the revised manuscript.*

**Comment 3:** Authors should check the places where they used “Figs” to refer to only a single figure throughout the manuscript.

***Response:*** *Thanks for reviewer’s valuable comments. We have revised it throughout the manuscript.*

**Comment 4:** In Additional file 6, authors included many unreferenced tables.

***Response:*** *I am very sorry for this confusion. We have revised it in the revision.*

**Comment 5:** Authors should make sure the gene names in Fig. 5b,e match the gene names included in Additional files 4 and 6.

***Response:*** *Thanks for reviewer’s valuable comments. KEGG pathway images (Fig. 5b, e) were generated with the KEGG Mapper Color Tool (*[*https://www.kegg.jp/kegg/mapper/color.html*](https://www.kegg.jp/kegg/mapper/color.html)*). KEGG pathway representation is an abstract network of gene products, and focuses on the network of gene products (Kanehisa et al, 2000 and 2004). The box in the KEGG pathway figure represents the gene product (below figure). Thus, a box may contain multiple genes in the KEGG pathway (below figure). We have added the gene product name and gene name in the* ***Additional files 5 and 8****.*

***References:***

*Kanehisa M, Goto S. KEGG: kyoto encyclopedia of genes and genomes[J]. Nucleic acids research, 2000, 28(1): 27-30.*

*Kanehisa M, Goto S, Kawashima S, et al. The KEGG resource for deciphering the genome[J]. Nucleic acids research, 2004, 32(suppl_1): D277-D280.*

**Comment 6:** Supplemental tables in Additional files 4 and 5 are mislabeled.

***Response:*** *I am very sorry for this mistake. We have revised it.*

**Reviewer #2**

**Comment 1:** Line 145, the 'is' should be 'was'. Line 151, the 'are' should be 'were'. Other parts of the manuscript also need to be checked.

***Response:*** *Thanks for reviewer’s valuable comments. We have revised it.*

**Comment 2:** Figures should be provided with vector images to avoid affecting clarity, as the current TIFF format lacks clarity.

***Response:*** *Thanks for reviewer’s valuable comments. We have revised it.*

**Comment 3:** Based on your findings, what potential impacts will it have on future animal husbandry or animal feed? Please explain.

***Response:*** *Thanks for reviewer’s valuable comments. We have added it as reading “These findings shed new light on nutrient–microbiome–host interactions in the early life. To our knowledge, this study is the first to demonstrate that early-life ruminal microbiome-derived IAld and PGD2 are effective promoters of rumen development, which provide new insights* *into research and application of novel feed additive in young ruminants. More importantly, we also offer first direct evidence for link specific ruminal strains and their downstream metabolites to host rumen wall physiology, which is one key to open the “black box” of rumen” in the revised manuscript* ***(Page 39-40, Line 533-540)****.*

**Third round of review**

**Reviewer 1**

The authors have addressed all my comments.

**Reviewer 2**

The manuscript is improved, but there are still many places in the manuscript that contain confusing grammar.

Line 206, 274, the ‘are’ should be ‘were’. Line 241, 259, the ‘have’ should be ‘had’, and so on. All sentences in the Results section should be in the past tense.

**Reviewer 3**

The authors have sufficiently addressed the previous comments of Reviewers 1 and 2.

**Authors Response**

**Point-by-point responses to the reviewers’ comments:**

**Reviewer #2:**

The manuscript is improved, but there are still many places in the manuscript that contain confusing grammar.

Line 206, 274, the ‘are’ should be ‘were’. Line 241, 259, the ‘have’ should be ‘had’, and so on. All sentences in the Results section should be in the past tense.

*Response: We have addressed all the grammar issues.*
